# Development of a long term, *ex vivo*, patient-derived explant model of endometrial cancer

**DOI:** 10.1371/journal.pone.0301413

**Published:** 2024-04-18

**Authors:** Hannah van der Woude, Khoi Phan, Diane N. Kenwright, Louise Goossens, Kathryn Elizabeth Hally, Margaret Jane Currie, John Kokkinos, George Sharbeen, Phoebe A. Phillips, Claire Elizabeth Henry

**Affiliations:** 1 Department of Obstetrics, Gynaecology and Women’s Health, University of Otago, Wellington, New Zealand; 2 Department of Pathology and Molecular Medicine, University of Otago, Wellington, New Zealand; 3 Medical Photography, Capital, Coast and Hutt Valley, Wellington, New Zealand; 4 Department of Surgery and Anaesthesia, University of Otago, Wellington, New Zealand; 5 Department of Pathology and Biomedical Science, University of Otago, Christchurch, New Zealand; 6 Pancreatic Cancer Translational Research Group, School of Biomedical Sciences, Lowy Cancer Research Centre, UNSW Sydney, Sydney, Australia; University of Michigan Medical School, UNITED STATES

## Abstract

Incidence of endometrial cancer (EC) is rising in the developed world. The current standard of care, hysterectomy, is often infeasible for younger patients and those with high body mass index. There are limited non-surgical treatment options and a lack of biologically relevant research models to investigate novel alternatives to surgery for EC. The aim of the present study was to develop a long-term, patient-derived explant (PDE) model of early-stage EC and demonstrate its use for investigating predictive biomarkers for a current non-surgical treatment option, the levonorgestrel intra-uterine system (LNG-IUS). Fresh tumour specimens were obtained from patients with early-stage endometrioid EC. Tumours were cut into explants, cultured on media-soaked gelatin sponges for up to 21 days and treated with LNG. Formalin-fixed, paraffin embedded (FFPE) blocks were generated for each explant after 21 days in culture. Tumour architecture and integrity were assessed by haematoxylin and eosin (H&E) and immunohistochemistry (IHC). IHC was additionally performed for the expression of five candidate biomarkers of LNG resistance. The developed *ex vivo* PDE model is capable of culturing explants from early-stage EC tumours long-term (21 Days). This model can complement existing models and may serve as a tool to validate results obtained in higher-throughput *in vitro* studies. Our study provides the foundation to validate the extent to which EC PDEs reflect patient response in future research.

## Introduction

Endometrial cancer (EC) is the most common gynaecological malignancy in the developed world but remains significantly under-researched [1–3]. The incidence of EC is rising and, although traditionally viewed as a post-menopausal disease, this rise is particularly evident in the pre-menopausal population. The reason for this is multifactorial and risk factors include parity later in life, lower parity, increased lifespan, polycystic ovarian syndrome, lynch syndrome, and perhaps most notably, obesity [4–7]. Of the four histological subtypes, endometrioid is the most common EC, occurring in 75–80% of cases [8], and is the subtype associated with high body mass index (BMI) [6]. Total hysterectomy is the recommended treatment for all stages of EC including pre-malignant endometrial hyperplasia, with later stages often also receiving pelvic lymph node dissection and bilateral salpingo-oophorectomy. The trend towards being diagnosed at a younger age and the increased surgical risk for those with a high BMI necessitates research into fertility-preserving, conservative management options for endometrioid EC (EEC).

Around 90% of novel therapeutics fail in randomised control trials [9]. Up to 50% of these failures are due to lack of clinical efficacy, which can be interpreted as a lack of pre-clinical models that accurately reflect *in vivo* response [9,10]. Given its ability to mimic the 3D multicellular architecture of human disease, the *ex vivo* patient-derived explant (PDE) culture technique has recently emerged as a powerful preclinical platform for cancer research [11]. PDEs are typically small (1–3 mm in diameter) pieces of fresh tumour that are cultured without disintegration into the tissue’s individual cellular components. Although methods of PDE culture vary, these models appear to have strong clinical applicability [12], and have shown promise in correlating with patient outcomes and predicting drug resistance in non-small cell lung carcinoma, gastric and colon cancer[13]. Advantages of PDE models over other forms of 3D models such as organoids is that the PDE model retains the native 3D tissue architecture and can therefore account for spatial and microenvironmental effects such as the effect of stromal and tumour-associated immune cells [14]. An *ex vivo* PDE model for EEC could prove beneficial for fast tracking novel, conservative (surgery-sparing) therapeutics from conception to clinical trial.

Radiotherapy and the levonorgestrel-(LNG) releasing intra-uterine system (IUS) are the only non-surgical treatments available for EEC patients. LNG-IUS, also known as Mirena®, has traditionally been used as a long acting, reversible contraceptive. As an EC therapy, LNG-IUS is only feasible for early-stage cases of endometrioid histology and pre-malignant endometrial hyperplasia, both of which currently carry the primary treatment modality of total hysterectomy. LNG is a synthetic progestogen which mimics the naturally occurring hormone, progesterone. In the context of cancer therapeutics, LNG counterbalances estrogen-driven cell proliferation, which is often the molecular mechanism underlying EEC. However, response rates for treatment of early-stage EEC with LNG-IUS in the literature vary between 40%-80% [15,16]. Despite the variability in reported response rates, there are currently no approved biomarkers predicting LNG-IUS response. As a result, patients fitted with the LNG-IUS undergo biopsies every 3–6 months to assess treatment efficacy in real time [17]. The invasive nature of these biopsies creates an urgency for research into novel, clinically useful biomarkers to predict response to LNG-IUS therapy [18,19]. The ability to predict LNG-IUS resistance prior to administration would ensure this treatment option is only administered to patients who would benefit from it. We propose that the development of a clinically and biologically relevant model of EEC would provide a robust platform for assessing both biomarkers of LNG-IUS response and investigation of conservative treatment for EEC.

## Materials and methods

Information regarding the materials and antibodies used can be found in S1 and S2 Tables, respectively.

### Cohort

**Fig 1:**
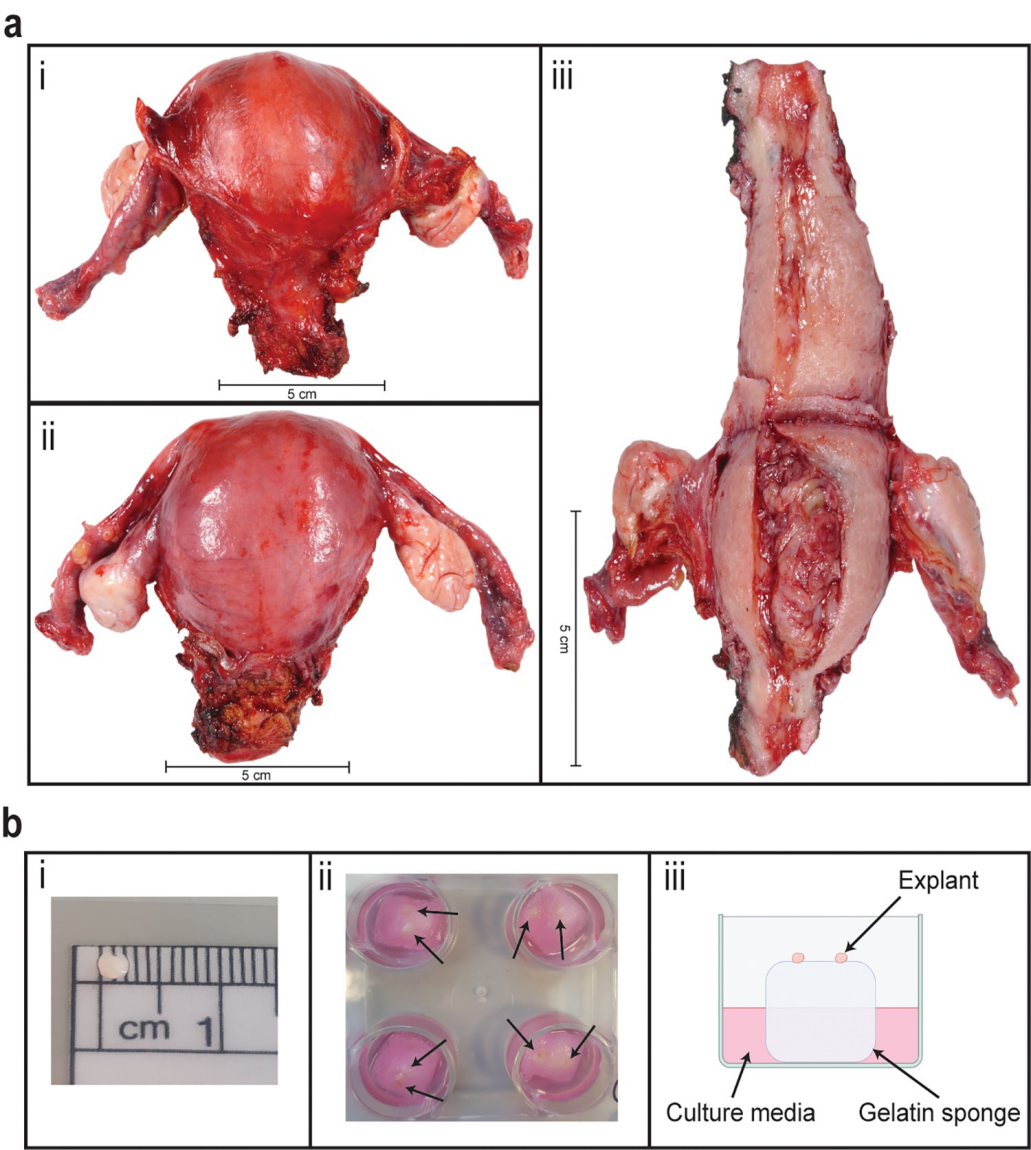
*Ex vivo* cell culture set up. (A) Example of a uterus used in the study, photographed from i) anterior, ii) posterior, and iii) open anterior view. Scale bars represent 5 cm. (B) Cell culture methodology. i) Explant tissue size. ii) Example of plate containing 2x explants per well. Black arrows point to individual explants sitting on top of a gelatin sponge. iii) Schematic of cell culture technique.

All patients undergoing hysterectomy after a diagnosis of early-stage EEC at Wellington Regional Hospital between 6^th^ July 2021 and 21^st^ June 2022 were invited to participate in the study and provided informed, written consent. Ethical approval was obtained from the Human Disability Ethics Committee (HDEC) (15/CEN/143) and the University of Otago Ethics Committee (H20_002). Consultation was undertaken with Research Advisory Group–Māori (RAG-M). All research was performed in accordance with the relevant guidelines/regulations and the Declaration of Helsinki. Authors had access to identifiable information from participants during the data collection in order to obtain final pathology reports. Following excision during surgery, the whole uterus was opened along the sagittal plane and the tumour was inspected by a pathologist and photographed by a medical photographer (L.G) (Fig 1A). A section of fresh tumour tissue, excess to diagnosis, was excised from the uterus into cold phosphate buffered saline (PBS).

### *Ex vivo* culture

Culture methods closely follow those of Kokkinos and colleagues for the development of an *ex vivo* PDE model of pancreatic cancer [20]. Prior to tissue collection, haemostatic gelatin sponges (Johnson & Johnson; NJ, USA) were dipped in culture media and placed into a well of a 24-well plate. Culture media containing Gibco™ DMEM/F12 plus Glutamax (Thermo Fisher Scientific; MA, USA), 10% Gibco™ fetal bovine serum ((FBS) New Zealand origin, Fisher Scientific; MA, USA), 1% Gibco™ penicillin/streptomycin (Fisher Scientific; MA, USA), 0.01 mg/mL hydrocortisone (Sigma-Aldrich; MO, USA) and 0.01 mg/mL human insulin (Sigma-Aldrich; MO, USA) was pipetted into the well so that the sponge was half-soaked in media (500 μL). Plates were warmed in a 37°C incubator with 5% CO_2_ for at least 1 h until required. The tumour resection was cut manually into explants with diameter ranging from 1–3 mm within 2 h of uterus excision. For each treatment, up to four explants across two gelatin sponges (two explants per sponge) were used for *ex vivo* culture at 37°C with 5% CO_2_. Additionally, a piece of tumour tissue was immediately fixed in 4% paraformaldehyde (PFA, Alfa Aesar; MA, USA) at 4°C for 24 h and designated as the Day 0 control. Explants from each tumour sample were treated daily on weekdays (Monday-Friday) with 4 ng/mL or 4 μg/mL LNG (Sapphire Bioscience; NSW, Australia) or 0.08% dimethyl sulfoxide (DMSO) (vehicle control), or normal culture media. The lower amount of LNG (4 ng/mL) was approximated to mimic exposure of the endometrium to LNG released from the LNG-IUS *in vivo* (S1 Methods), and the higher amount was 1000x in excess of the calculated biologically relevant dose.

For *ex vivo* model validation, explants were removed from the sponge at days 7, 14 and 21, fixed as described above and maintained in 70% ethanol until tissue processing. One formalin fixed paraffin embedded (FFPE) block was generated per explant and cut into 4 μm serial sections. For LNG treatment and biomarker investigation, FFPE blocks were generated only for explants cultured for 21 days. For examination of proliferation using Bromodeoxyuridine (BrdU), 10 μM BrdU substrate (Thermo Fisher Scientific; MA, USA) was added to explant culture media 24 h prior to fixation at Day 21. Haematoxylin and Eosin (H&E) staining was carried out and explant architecture was assessed visually by a trained gynaecological histopathologist (D.K).

### Immunohistochemistry

Immunohistochemistry (IHC) for *ex vivo* model validation was carried out using the Ventana Benchmark Ultra staining platform using sections from explants cultured for 7, 14 and 21 days on the following antibodies: Ki67 (Ventana; AZ, USA); Phosphohistone H3 (PHH3; Cell Marque; CA, USA) and Progesterone Receptor (PR; Roche Diagnostics; Switzerland). Ki67 staining was performed by Wellington Southern Community Laboratories while PHH3 and PR staining was performed by MedLab Central (Palmerston North, New Zealand). IHC for Cleaved Caspase-3 (CC3; Cell Signalling Technology; MA, USA) was carried out on a Leica Bond RX machine. Bromodeoxyuridine (BrdU) staining and terminal deoxynucleotidyl transferase dUTP nick end labelling (TUNEL) were carried out manually according to the manufacturer’s instructions. Antibody information is listed in S2 Table. BrdU and TUNEL methods are outlined in S2 and S3 Methods, respectively. Representative images of BrdU staining were taken per explant using an Olympus BX53 light microscope. Representative images of TUNEL staining were taken per explant using an Olympus CKX53 microscope with X-Cite series 120Q for fluorescence illumination. The imaging was performed with Olympus UIS2 fluorescence mirror unit microscope attachments for blue (DAPI) and green (TUNEL) excitation. All images were taken using Olympus cellSens software version 2.3. TUNEL staining was semi-quantified using QuPath software version 0.3.2 (Queen’s University; Ireland). The positive cell detection function was used to identify the number of cells co-expressing TUNEL (green) and DAPI (blue) as a percentage of all cells expressing DAPI.

### Candidate predictive biomarkers of LNG resistance

IHC was performed on Day 0 tissue and explant tissues which had been cultured in either 4 ng/mL or 4 μg/mL LNG media, 0.08% DMSO or untreated culture media for 21 days. Because we would expect a response after 21 days of LNG treatment if the tumour was an inherent responder, tumours that produced at least one explant showing minimal sign of response (>90% viable) after 21 days of the highest LNG treatment (4 μg/mL) were deemed “quasi-resistant”. Of the quasi-resistant tumours, explants were selected for biomarker investigation using IHC from the remaining treatment groups based on having a combination of a high epithelial tumour compartment and a high proportion of viable tissue of the replicates within each treatment group. The viability of the epithelial tumour compartment of the selected explants were judged by a histopathologist (D.K) based on H&E staining.

Five proteins were selected from previous biomarker discovery work in our laboratory which were demonstrated to be consistently upregulated on or within EEC cells that were resistant to LNG [21,22]. The following proteins were investigated for their expression pattern in explants by IHC: Special AT-rich sequence-binding protein 2 (SATB2), kruppel like factor 4 (KLF4), monoamine oxidase A (MAO-A), aldehyde dehydrogenase 1 family member A1 (ALDH1A1) and cluster of differentiation 80 (CD80) (S2 Table). SATB2 staining was performed on a Ventana Benchmark Ultra staining platform. KLF4, MAO-A, ALDH1A1 and CD80 were diluted in Bond Primary Antibody Diluent (Leica Biosystems; Germany) and run on a Leica Bond RX. Explants with a high degree of non-specific staining were excluded from downstream analysis.

Three authors (H.V, C.H, D.K) including one gynaecological histopathologist (D.K), separately reviewed IHC staining of the candidate biomarkers under a light microscope and scored expression on a scale of 0–3 (0, no expression; 3, high expression) based on the proportion of positive tumour cells within the explant, and the intensity of staining combined.

## Results

### Development of a long-term, *ex vivo* culture model of endometrial cancer

**Fig 2:**
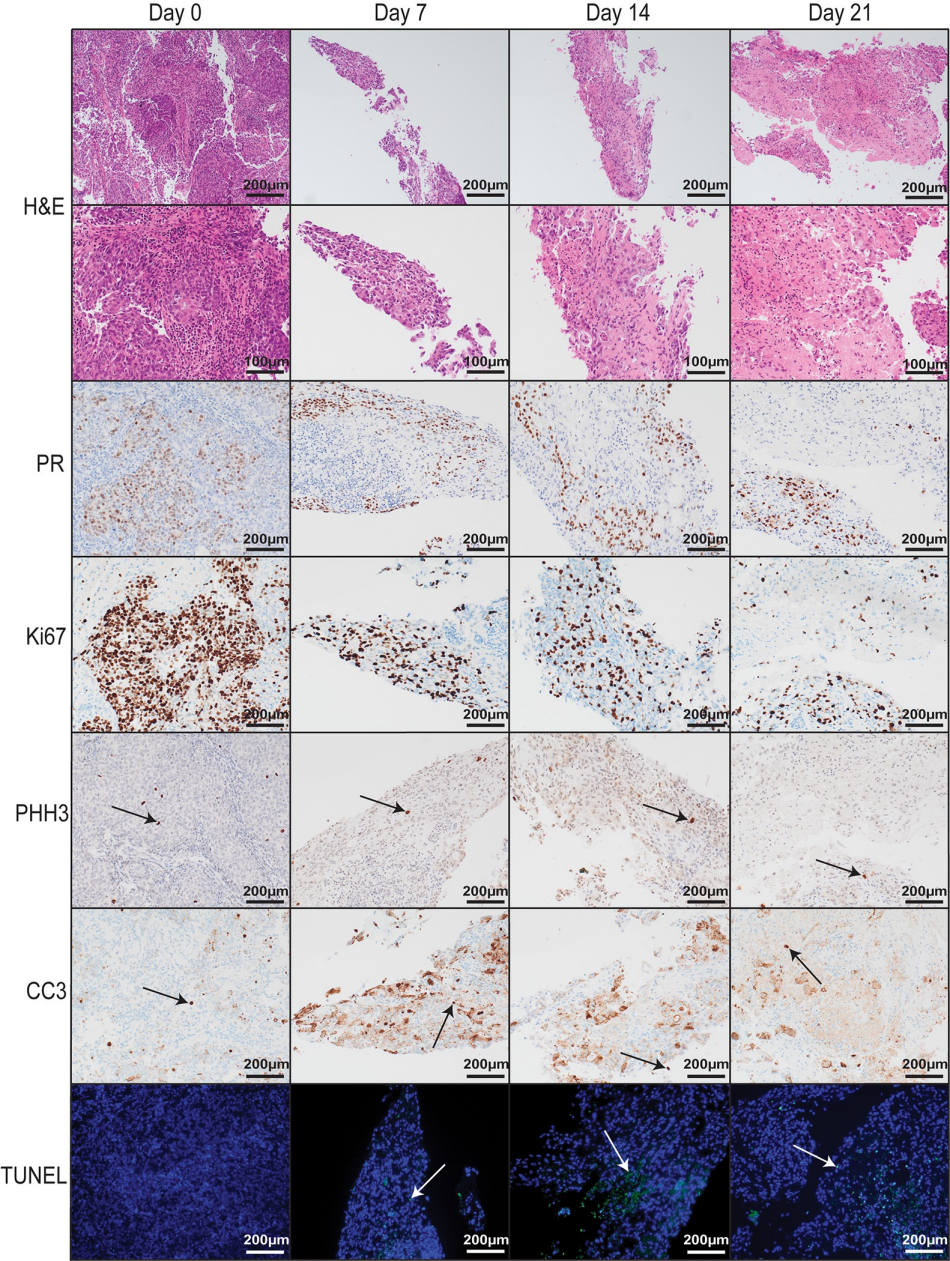
Representative images from Patient 1 of H&E and IHC from Day 0 to Day 21 in *ex vivo* culture. H&E and IHC for PR expression, proliferation (Ki67, PHH3) and apoptosis (CC3, TUNEL). In the TUNEL row, blue staining reflects DAPI indicating intact DNA and green reflects TUNEL, indicating apoptosis. H&E, haematoxylin and eosin; IHC, immunohistochemistry; PR, Progesterone Receptor; PHH3, Phosphohistone H3; CC3, Cleaved Caspase 3; TUNEL, Terminal deoxynucleotidyl transferase dUTP nick end labelling; DAPI, 4′,6-diamidino-2-phenylindole.

**Fig 3:**
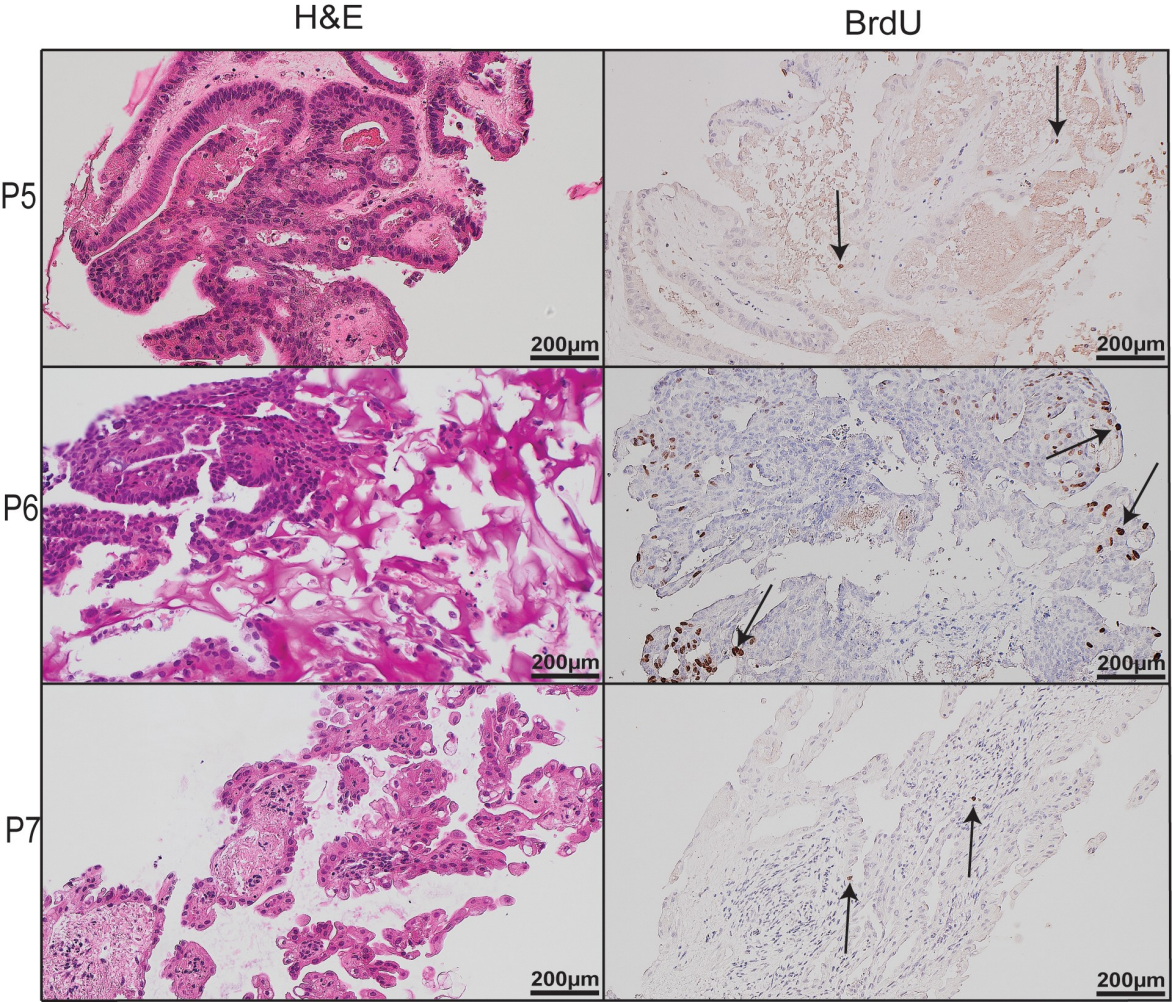
BrdU staining. Representative images from three patients (P5, P6, P7) on untreated explants after 21 days in *ex vivo* culture with the addition of BrdU (BrdU was added to culture medium 24 h before fixing explants). H&E, Haematoxylin and Eosin; BrdU, Bromodeoxyuridine / 5-bromo-2’-deoxyuridine.

Ten patients with EEC were recruited into this study. Their demographics and clinicopathological characteristics are listed in Table 1. Of the patients included in the study, two (Patients 1 and 3) had been previously treated with LNG-IUS for early-stage EC. Tumour was obtained from the uterus of patients undergoing hysterectomy (Fig 1A). Resected tumour tissue was transported to the laboratory in cold PBS and cultured on a media-soaked gelatin sponge as explants 1–3 mm in diameter for up to 21 days (Fig 1B). H&E staining and PR expression in explants from Day 0 (uncultured), Day 7, Day 14, and Day 21 showed explant architecture and tumour integrity was maintained over time in the *ex vivo* PDE model (Fig 2). A time course of explants from three other patients can be viewed in the supplementary material (S1–S3 Figs). Similarly, the proliferative capacity of tumour explants was maintained for the length of the culture period, as shown by positive IHC staining for two independent proliferation markers, Ki67 and PHH3 (Fig 2), although expression of these markers is lower in cultured explants compared to uncultured tissue (Fig 2 and S1–S3 Figs). The proportion of apoptotic cells (identified by CC3 and TUNEL) in explants from Patient 1 increased compared to uncultured tissue, however the rate of apoptosis remained stably elevated throughout the culture period (Fig 2). This pattern was also observed in Patient 8 explants (S1 Fig), while in Patient 9 explants, CC3 staining did not appear to change in cultured versus uncultured tissue (S2 Fig). *De novo* proliferation status was confirmed by positive nuclear BrdU staining at Day 21, indicating continued proliferation after 21 days in culture (Fig 3).

**Table 1:** Patient characteristics.

Total	10
**Age (years)**	
Median age	61.5
Age range	46–76
**BMI**	
Median BMI	30.5
BMI range	26–49
**Histopathology Diagnosis**	
Endometrioid	10
Grade 1	4
Grade 2	1
Grade 3	2
Stage Ia	2
Stage Ib	3
Stage II	2
Stage IIIa	3
**Molecular Profile**	
MMRD	1
P53 abnormal	1
**Mirena *in situ***	2

BMI, body mass index; MMRD, mismatch repair deficient.

### LNG treatment of explants

**Fig 4:**
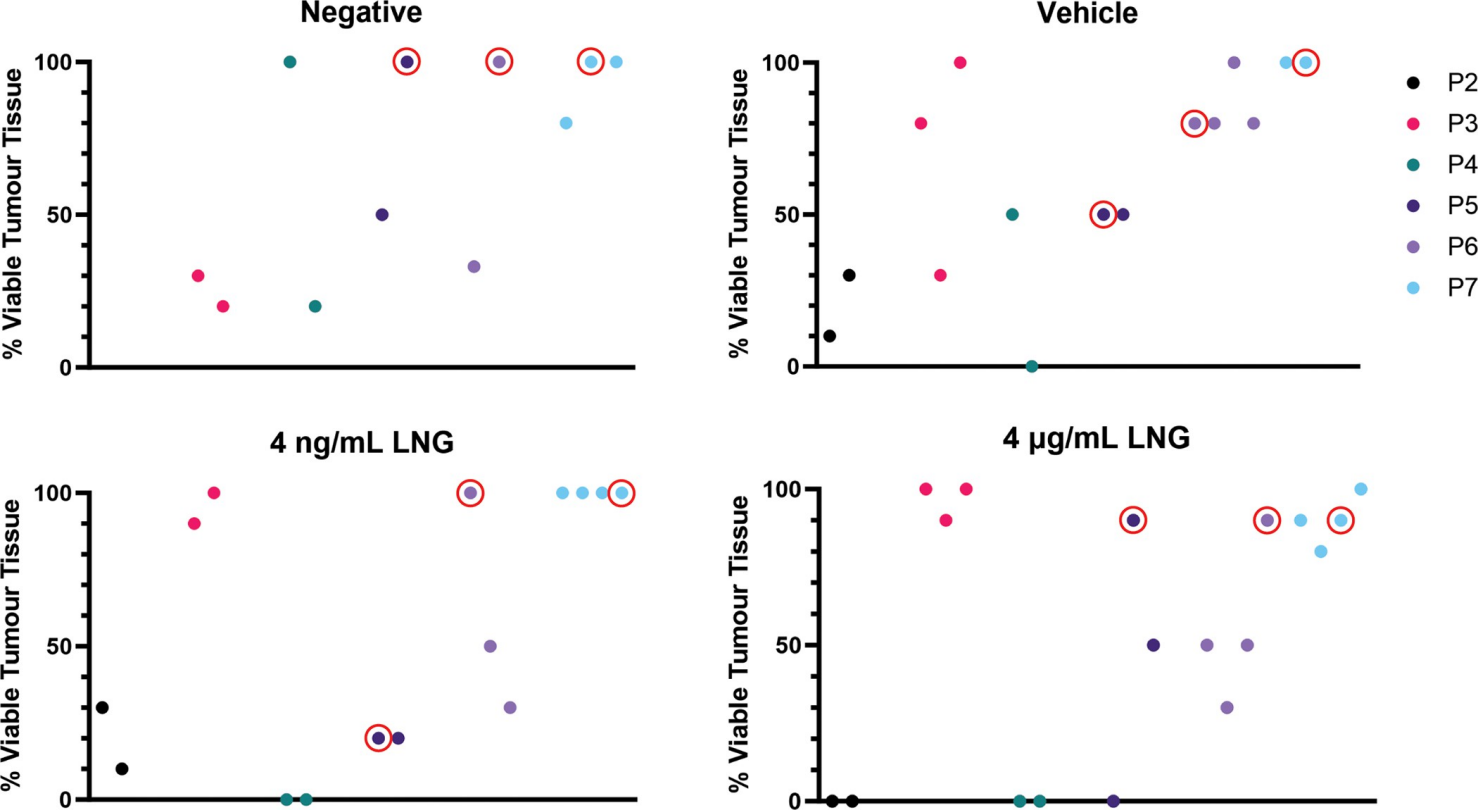
Variability in viability of the tumour tissue within explant replicates after 21 days in ex vivo culture. Explants from six patients were treated with LNG for 21 days in the *ex vivo* culture model. A histopathologist assessed the viability of the tumour tissue within each explant based on H&E stains. Patients 2 (P2) and 4 (P4) were excluded from downstream analysis due to low viability of 4 μg/mL LNG explants, and Patient 3 (P3) was excluded due to having a small epithelial tumour compartment. Red circles indicate the explants selected for downstream assessment. Explants were selected based on high viability and large epithelial tumour compartment. Neg, negative control (culture media alone); Vhc, vehicle control (culture media plus 0.08% DMSO); LNG, levonorgestrel (4 ng/mL or 4 μg/mL).

After 21 days, explants were fixed, embedded and stained with H&E (S4 Fig) and assessed for viability (S5 Fig). High variability was observed in the viability of the explants, even in explants from one tumour sample cultured under the same conditions (Fig 4). Due to this variability, it was difficult to determine which patients were intrinsic responders to LNG treatment using this model. Therefore, the tumours that produced an explant with high tumour viability (>90% viable) after 21 days of treatment with 4 μg/mL LNG were deemed “quasi-resistant”. i.e. the explants treated with LNG for 21 days were examined by H&E stain and the viability of the epithelial compartment was assessed by our histopathologist (D.K.). If the viability was more than 90% then that tumour was considered likely to be resistant to LNG and was therefore quasi-resistant. The viabilities of explants selected for downstream IHC assessment are available in S3 Table. Patients 2 and 4 were excluded due to low viabilities, and Patient 3 was excluded due to a low volume of tumour tissue within the explants. Explants from Patient 1 were used to validate the *ex vivo* model prior to commencement of LNG treatment and thus were not included in the downstream LNG analysis.

**Fig 5:**
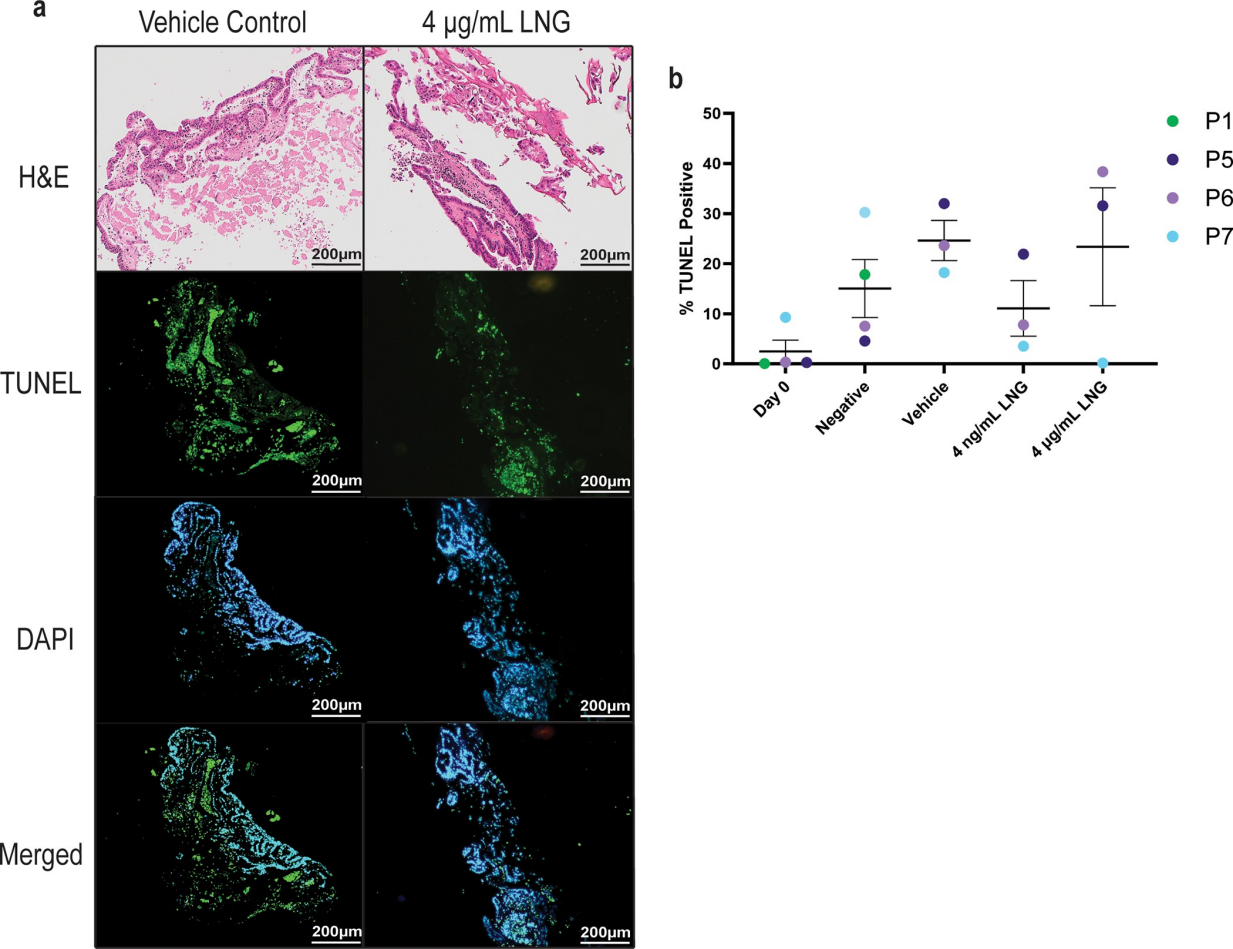
Assessment of apoptosis in LNG quasi-resistant explants. Visualised using TUNEL, in endometrial cancer explants after 21 days in *ex vivo* culture. (A) Representative images of TUNEL. Explants from Patient 5 which were treated with either vehicle control (culture media plus 0.08% DMSO) or 4 μg/mL LNG for 21 days and stained with TUNEL (green) and DAPI (blue). (B) TUNEL positive cells as a percentage of all cells from explants treated with either culture media (Negative), DMSO (Vehicle), 4 ng/mL LNG or 4 μg/mL LNG. Plot shows mean and standard error of the mean (SEM) bars. A mixed effects analysis showed no significant difference in TUNEL positivity between treatment groups. LNG, Levonorgestrel; H&E, Haematoxylin and Eosin; TUNEL, Terminal deoxynucleotidyl transferase dUTP nick end labelling; DAPI, 4′,6-diamidino-2-phenylindole.

TUNEL staining was performed on explants from quasi-resistant tumours to assess apoptosis (Fig 5A) and further demonstrated individual variability of response (Fig 5B). Additional representative images of TUNEL positivity staining, as well as a visual guide for TUNEL positivity assessment can be found in S6 Fig. Overall, apoptosis was not significantly increased in the quasi-resistant tumours that were treated with LNG compared to untreated (negative) and vehicle controls. Although apoptosis appeared to increase during *ex vivo* culture when comparing uncultured (Day 0) controls to explants cultured for 21 days, the majority of the tissue (>55%) was still viable after 21 days (Fig 5B).

**Fig 6:**
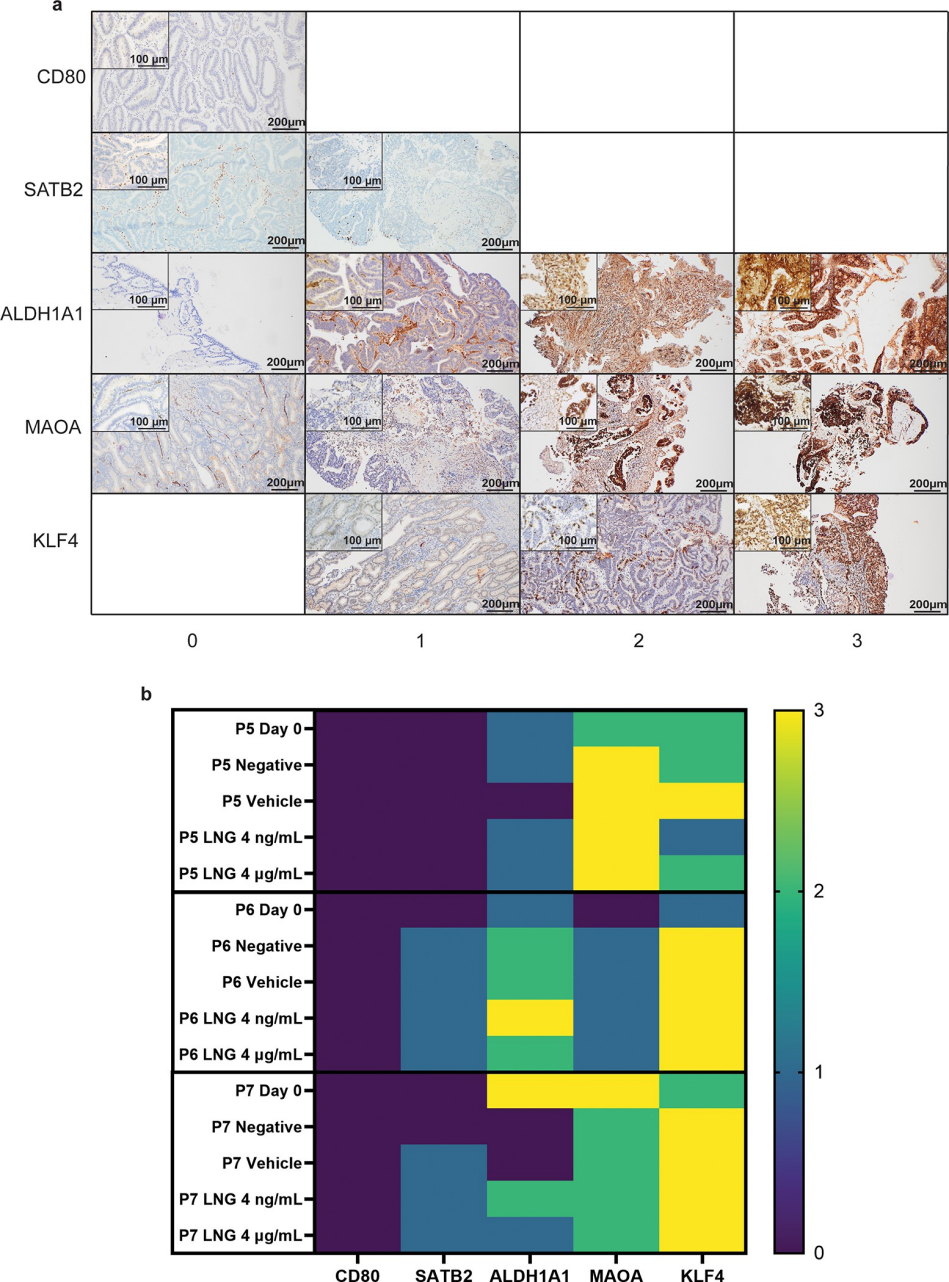
Candidate biomarker immunohistochemical analysis. Day 0 tumour tissue samples were taken on the day of surgery and tumour explants were fixed after 21 days of culture in culture media alone (Negative), culture media plus 0.08% DMSO (Vehicle), or LNG (4 ng/mL, 4 μg/mL). IHC stains were scored by three independent authors (H.V, C.H, D.K) on a four-point scale of 0–3 based on the proportion of positive tumour cells within the explant and the staining intensity. (A) Representative images of the 0–3 point scale for all markers. (B) Heatmap of the median stain score for each biomarker against treatment for three patients. LNG, Levonorgestrel; IHC, Immunohistochemistry.

On Day 21, the most viable explants from quasi-resistant tumours were fixed and stained immunohistochemically for their expression of five candidate proteins previously identified in a 2D model of EEC as biomarkers of resistance to LNG treatment in our laboratory [21,22]. The candidate biomarkers were examined in the current study to demonstrate the feasibility of using this PDE model for biomarker validation. Protein expression was scored by three observers on a scale of 0–3 (Fig 6A) and the median score across the three observations was taken (Fig 6B). Necrotic and stromal regions of tissue were ignored. There was minimal staining of CD80 and SATB2 in the tumour compartment (Fig 6B). KLF4 had the highest degree of staining of the candidate biomarkers examined, and MAOA and ALDH1A1 were moderately expressed. There were no consistent observations made across treatment conditions.

## Discussion

In this study, we developed a long-term (21 day), *ex vivo*, patient-derived explant (PDE) culture model of early-stage EEC and utilised it to test the current non-surgical treatment, LNG-IUS, and the expression of candidate biomarkers of LNG resistance. Patients who are fitted with an LNG-IUS for EEC treatment are generally followed up on after 3–6 months [17]. The model established here can be maintained for 21 days. It therefore has the potential to model response to therapeutics in a way that more closely resembles *in vivo* biology. Although the long-term culture of early-stage EEC PDEs presented here does not have a 100% success rate, there is a need for long-term cultures which demonstrate an effect of long-term surgery-sparing treatments such as the LNG-IUS. Shorter term cultures, which may have a higher success rate, are unable to provide information on these long term changes post treatment. In this study we demonstrated the preservation of native tumour 3D architecture and proliferative capacity on unmanipulated EEC explants for up to 21 days in culture, although markers for apoptosis tended to be increased in cultured explants compared to uncultured (Day 0) controls. Tumours with explants that survived 21 days of 4 μg/mL LNG treatment were deemed quasi-resistant to LNG. These tumours could not unequivocally be considered resistant because the length of time given for the tumour to respond *in vivo* is up to 6 months [17] and the length of our culture was three weeks. Quasi-resistant tumours were investigated for their expression of candidate biomarkers of LNG resistance across treatment groups. CD80 and SATB2 were minimally expressed across the explants analysed, while MAOA and ALDH1A1 showed moderate staining, and KLF4 expression was high. This model could therefore be used to validate 2D model-based biomarker discovery studies.

PDE models have been established in a range of cancers including pancreatic, prostate, breast and ovarian [20,23–26]. Recently, a short-term (48 h) PDE model of EC was employed to explore tumour response to therapeutics [27]. In lieu of gelatin sponges, authors utilised Millipore PVDF filter supports onto which tumour explants were placed. Explants maintained native architecture as well as intrinsic cellular parameters after 48 h in culture, demonstrated by H&E and IHC staining for proliferation and death markers. The model was also used to assess explant sensitivity to standard-of-care chemotherapeutics carboplatin and paclitaxel, and results corresponded with the expected proportion of responders to these treatments [27]. Previous evidence showed that *ex vivo* models can be used to predict patient response to therapeutics [28], and the research by Collins *et al* demonstrated that EC PDE models also have this potential in the short term (less than 48 h) [27]. We demonstrate that these cultures can be extended to 21 days in some cases. In our PDE model, proliferating cells in the S phase of the cell cycle are present after 21 days, evidenced by BrdU staining (Fig 3). When comparing with Ki67, which enables visualisation of cells in all phases of the cell cycle except resting (G0), there was a slight reduction of Ki67 expression in cultured compared to uncultured tissue, but a large proportion of proliferating cells were maintained to Day 21 (Fig 1). There was increased apoptosis in cultured explants compared to uncultured tissue, however apoptosis did not increase noticeably between day 7 and 21 (Fig 1 and S1–S3 Figs).

We found that there was significant heterogeneity in survival of explants using the PDE model outlined in this study which resulted in fewer patients being included in the final analysis, and which made interpreting results difficult. The variable explant viability was likely due to a combination of sampling and handling techniques, the low grade and stage of the tumours, as well as tumour heterogeneity, which has been noted previously with organoid viability [29]. For example, tumour samples were taken fresh at the time of hysterectomy. Some patients had minimal or non-pedunculated tumours; some tumours were often hard to see on a fresh (versus formalin fixed) specimen. This may have contributed to the amount of solid tumour removed in the process of sample retrieval. This necessitated the generation of multiple explants per treatment which may impose practical challenges in a high-throughput laboratory or with small volume samples. EEC is typically a slow-growing cancer and resecting early-stage tumours could have led to inconsistent viability of the explants due to its non-aggressive nature. Moreover, explants from early-stage tumour are likely to contain non-cancerous epithelium and stromal cells. While we were interested primarily in the tumour compartment, these other cell types comprise the tumour microenvironment and could have had varying impacts on explant survival. Other cancer types where the long-term PDE model has been established, such as pancreatic [20] and ovarian [26] cancer, are highly aggressive and have high stromal content compared to EEC, which tends to be quite mucinous. As stroma are known to provide signals aiding tumour growth, invasion, and metastasis [30], PDEs from pancreatic or ovarian tumour would be more likely to survive *ex vivo*. This explains the lower degree of apoptosis observed in explants generated from pancreatic cancer compared to early-stage EEC [20]. Notwithstanding, the current study provides a method to maintain EEC PDEs that survive 21 days in culture in some cases. As stated for PDE models in ovarian cancer, one of the major limitations of this model is the small volume of tumour necessary to culture the tissue. This means that the explant itself is going to be only a small representation of the heterogeneity of the whole tumour [31]. High tumour heterogeneity, where very similar regions of tumour tissue contain tumour cell clones with differing resistance to LNG, may also have affected our explant viabilities and supports the notion that multiple explants from a single patient should be generated. Although this is a low-throughput setup, the EEC PDE model poses advantages over alternative higher throughput options like organoids, the greatest of which is a better reflection of *in vivo* tissue architecture. The PDE model could be used to complement high-throughput discovery work conduced in organoid models as well as 2D cell line models.

Most PDE cultures in the literature are short-term (between 48 h and 7 days), although we and others have developed long-term cultures (up to 30 days) [24,26,28]. Methods to increase nutrient and oxygen diffusion to maintain explant survival over time vary. For example, Karekla *et al* placed fragments of non-small cell lung carcinoma tissue onto a culture insert disc floating on cell culture media [28], whereas Abreu *et al* placed ovarian carcinoma PDEs in a flask with constant orbital shaking [26]. The most common technique, also utilised in the current study, is the use of a haemostatic gelatin sponge, which acts as the mediator between nutrient media and tissue [20,24].

As our aim was to establish a PDE model for testing surgery-sparing treatments, which are only considered for early-stage EEC, we focussed our sampling on stage 1 EEC (stage 1A and stage 1B). It is worth noting that although in some cases the post-surgical pathology assessment increased the patient’s grade or stage to outside these parameters, all patients had a pre-surgical assessment of early stage and/or low grade which would qualify them for LNG treatment. Two patients had been previously treated with LNG-IUS for EEC. One (Patient 3) was excluded from biomarker analysis due to a low amount of tumour within the explants, possibly the result of LNG-IUS treatment, and explants from the other (Patient 1) were used to validate the *ex vivo* model prior to commencement of LNG treatment.

Early-stage EEC is often driven by estrogen stimulation with deficient progesterone opposition [32,33]. As estrogen was not added to the culture media, this molecular driver of cell proliferation could have been lost and would explain the drop in Ki67 expression and increase in apoptosis from uncultured tumour compared to cultured explants. As we were assessing LNG, which is a progestogen, it was not feasible to include estrogen in the culture media as it could have interfered with LNG treatment. Due to the small size of the explants that represent different pieces of the original excised tissue, it was difficult to determine whether there were natural pockets of non-proliferating cells present within explants or whether cell proliferation was affected by culturing. We observed CC3 expression across all timepoints of *ex vivo* culture in Patient 1 –including at Day 0, and both CC3 and TUNEL staining was higher in cultured explants compared to uncultured tissue. This is unsurprising due to the significant environmental changes that the tissue experiences when being cultured using an *ex vivo* method, however it is a limitation that could be addressed in the future. In the other three time courses presented in the supplementary material there is variability in CC3 expression. Patient 8 displayed a similar profile to Patient 1 (S1 Fig), Patient 9 had CC3 positive cells at Day 0 which were maintained at each timepoint (S2 Fig), and Patient 10 had very little CC3 staining at Day 0, a high degree of staining at Day 7 and then appeared to recover by Day 14 as the CC3 positivity decreased (S3 Fig). Based on this variance, it is likely that the generation of successful long-term PDEs for early-stage EEC is patient-dependent.

A clinical test for LNG resistance in the form of a biomarker would be hugely beneficial. The most recent clinical trial reported complete pathological response to LNG-IUS treatment for EEC of 42% [15]. This means that over half of the patients who receive LNG-IUS treatment are not likely to respond. Follow up appointments to assess response occur roughly every 3–6 months [17]. There are many challenges with the treatment pathway for patients undergoing conservative management of EEC. Patients undergo pipelle or hysteroscopy dilation and curettage (D&C) to sample the endometrium in order to assess response to therapy. Patients require two negative biopsies to confirm response to treatment. Anecdotally, we observed high non-attendance for pipelle biopsies, and frequent distress or anxiety around in-clinic pipelles. This route of sampling can also result in ‘insufficient tissue’; pathologists cannot determine whether there is simply not enough cancer because of the sample method, or because the cancer has regressed. Furthermore, LNG can have a metaplastic effect on endometrial cancer, making it difficult for pathologists to diagnose ‘treatment effect’ or regression in comparison to persistent disease. Women who do not respond to LNG-IUD experience a delay of effective treatment which can decrease survivability [34]. An approved biomarker for LNG response would ensure that only those likely to respond are offered this treatment, reducing avoidable cancer progression and increasing timely treatment of disease.

To demonstrate the practicality of the *ex vivo* PDE model generated in this study, LNG quasi-resistant EEC tumours which were treated and not treated with LNG were investigated for their expression of candidate biomarkers. The quasi-resistant state of the explants was reflected in Fig 5B, where the degree of apoptosis identified by TUNEL was not significantly increased in the LNG treated explants compared to the untreated (negative) and vehicle controls. Previous cell line work in our laboratory generated EEC cells resistant to LNG [21]. Ensuing whole genome RNA sequencing and bioinformatic analysis revealed a number of differentially expressed genes in LNG resistant compared to LNG sensitive cell lines [22]. Many of these proteins had not been previously investigated in the context of EEC. Five of these proteins were further investigated using the PDE model in the current study. There was low SATB2 and CD80 protein expression in the explants studied. As the explants used in the current study represent a sample of quasi-resistant tumours (non-responders) it would be interesting to assess SATB2 and CD80 expression in tissue from LNG responders to determine their clinical applicability as biomarkers of LNG response. The remaining three candidate biomarkers investigated are involved in maintaining cancer stem cells (CSC), which are known to be more invasive and treatment resistant than standard cancer cells [35–38]. ALDH1A1 is a cytosolic enzyme which catalyses the production of retinoic acid from retinal, and is part of the reaction series that clears ethanol from the liver [39]. ALDH1A1 overexpression can be oncogenic, and it is thought to exert this effect by maintaining the CSC population [40]. ALDH1A1 was variably expressed in the quasi-resistant explants examined and may hold value as a predictive biomarker for LNG resistance in EEC. MAOA is a mitochondrial enzyme with primary function in the brain due to its involvement in the catabolism of monoamine neurotransmitters [41,42]. As such it has been implicated in mental illness and stress [43,44], but it also has a role in cancer progression. For example, in a mouse model of prostate cancer *MAOA* knockout mice had reduced risk of invasive cancer and a reduced population of CSCs [45]. Expression of MAOA in most of the explants warrants further investigation of this candidate biomarker. KLF4 belongs to the zinc-finger protein family of transcription factors and has diverse functionality including cell cycle regulation, p53 regulation, and induction of pluripotent stem cells [46–48]. *KLF4* methylation has been investigated as a predictor of EC with increased CpG methylation being associated with increased risk of EC [49]. The consistent elevated expression of KLF4 in the explants examined combined with its expression in LNG-resistant endometrial cancer cell lines and primary cells [21] provides evidence for KLF4 as a potential biomarker for LNG resistance, and further investigation of this protein in a larger patient cohort is warranted. Encouragingly, ALDH1A1, MAOA and KLF4 were all expressed to some degree at Day 0 and after 21 days of both low and high doses of LNG treatment (Fig 6). This indicates that there is sustained expression of these markers long-term in tumours that are resistant to LNG treatment. Proteins that are contributing to LNG resistance would necessarily sustain their expression in order for resistance to persist. This provides further evidence for warranting closer examination of these candidate biomarkers in patient diagnostic biopsies [15,17,34].

The difficulties associated with both defining clinical LNG response and obtaining tissue biopsies for this PDE model outside of hysterectomy means that PDE models and other forms of *in vitro* models derived from this surgery are best used for studying mechanisms of LNG resistance in order to inform clinical decision-making/guidelines for recommending LNG-IUS to future patients. For personalising decision-making around proceeding with an LNG-IUS versus hysterectomy, a biopsy derived from a clinically routine pipelle targeting biomarkers, once established, would be more appropriate than a PDE.

This was a pilot study with the overall aim of developing and validating a PDE model of early-stage EEC. The sample size chosen was based on availability of patient tumour, length of time of the culture, and the survivability of the explants generated. The variability of response identified represents ‘real world’ patient variability and heterogeneity of the tumour. Further validation or subsequent studies that use the explant model would benefit from larger sample sizes. The explant model was used as a proof of concept to investigate LNG biomarkers and therefore the small sample size of the study limits the generalisability of the results. We recommend further investigation of these markers in larger cohorts, in particular in patient pipelle samples before and after treatment with the LNG-IUD. In this respect, the current study can be used to guide the feasibility of further examination of the candidate biomarkers examined. The development of long-term *ex vivo* PDE models has been successful in a number of cancer types. Overall, the benefits of using a biologically relevant PDE model can provide a useful tool to complement existing preclinical models. The amount of intra- and inter-patient variability in explant viability observed in the current study suggests that the use of a long-term *ex vivo* PDE explant culture for studying early-stage EEC would be best in low-throughput laboratories. Future studies could seek to improve this limitation. Although Collins *et al* reported that neither culture media nor serum supplementation had a significant effect on the viability of their EC explants [27], modifying culture conditions such as with the addition of growth factors used to sustain organoid cultures, or the EEC cell proliferation molecule estrogen could improve explant viability. An alternate culturing system with continuous media flow or orbital shaking could also be adopted [26]. Although we did not notice any major change in media colour over the weekend, PDE culture protocols for more aggressive cancers such as pancreatic recommend replenishing the nutrient media daily, which could improve the number of viable explants long-term [20]. Nevertheless, it is likely that this PDE model for early-stage EEC is better suited for shorter periods of time. Higher grade/stage EEC tumours, or those of a more aggressive histological subtype, such as serous, could be more likely to survive reliably long-term using the same methodology. However, this would not represent the disease state for LNG-IUD treatment. The model can be used to investigate protein expression for biomarker validation. Of the five candidate biomarkers of LNG resistance evaluated in this paper, KLF4 expression was high, while SATB2 and CD80 expression was low. Further investigation of ALDH1A1, MAOA, and particularly KLF4 in a patient cohort is warranted, and their involvement in maintaining cancer stem cell populations could lead to novel areas of investigation for non-surgical treatment of EEC and LNG resistance.

## Supporting information

S1 Table Materials information. (PDF)

S2 Table Antibody and immunohistochemistry information. (PDF)

S3 Table Explant viabilities from quasi-resistant tumours. (PDF)

S1 Fig Representative images from Patient 8 of H&E and IHC from Day 0 to Day 21 in *ex vivo* culture. H&E and IHC for PR expression, proliferation (Ki67, PHH3) and apoptosis (CC3). H&E, haematoxylin and eosin; IHC, immunohistochemistry; PR, Progesterone Receptor; PHH3, Phosphohistone H3; CC3, Cleaved Caspase 3.
(TIF)

S2 Fig Representative images from Patient 9 of H&E and IHC from Day 0 to Day 21 in *ex vivo* culture. H&E and IHC for proliferation (Ki67, PHH3) and apoptosis (CC3). H&E, haematoxylin and eosin; IHC, immunohistochemistry; PR, Progesterone Receptor; PHH3, Phosphohistone H3; CC3, Cleaved Caspase 3.
(TIF)

S3 Fig Representative images from Patient 10 of H&E and IHC from Day 0 to Day 21 in *ex vivo* culture. H&E and IHC for PR expression, proliferation (Ki67, PHH3) and apoptosis (CC3). H&E, haematoxylin and eosin; IHC, immunohistochemistry; PR, Progesterone Receptor; PHH3, Phosphohistone H3; CC3, Cleaved Caspase 3.
(TIF)

S4 Fig H&E stains. H&E was performed on tissue from three patients (P5, P6, P7) at Day 0 and Day 21 after culture with either vehicle control (culture media plus 0.08% DMSO) or LNG treatment. Scale bars represent 200 μm. H&E, Haematoxylin and Eosin; LNG, Levonorgestrel.
(TIF)

S5 Fig Viability based on H&E. Representative images depicting explants with tumour tissue that is 0%, 50%, 80% and 100% viable as identified by a trained gynaecological histopathologist.
(TIF)

S6 Fig TUNEL quantification. (A) Representative images for TUNEL quantification using QuPath software version 0.3.2. (B) Explants from Patients 6 and 7 which were treated with either vehicle control (culture media plus 0.08% DMSO) or 4 ug/mL levonorgestrel (LNG) for 21 days and stained with TUNEL (green) and DAPI (blue). Scale bars represent 200 μm.
(TIF)

S1 Methods Levonorgestrel calculation. (PDF)

S2 Methods BrdU immunohistochemistry. (PDF)

S3 Methods TUNEL immunohistochemistry. (PDF)

S1 Dataset (XLSX)

## References

[1] Sung H., et al., Global cancer statistics 2020: GLOBOCAN estimates of incidence and mortality worldwide for 36 cancers in 185 countries. CA: A Cancer Journal for Clinicians, 2021. 71(3): p. 209–249. doi: 10.3322/caac.21660 33538338

[2] Ministry of Health, New Cancer Registrations 2018. 2020.

[3] Henry C., et al., Beyond the numbers-understanding women’s experiences of accessing care for abnormal uterine bleeding (AUB): a qualitative study. BMJ Open, 2020. 10(11): p. e041853. doi: 10.1136/bmjopen-2020-041853 33203638 PMC7674079

[4] Wu Q.-J., et al., Parity and endometrial cancer risk: a meta-analysis of epidemiological studies. Scientific Reports, 2015. 5(1): p. 14243. 26373341 10.1038/srep14243 PMC4642705

[5] Sorosky J.I., Endometrial Cancer. Obstetrics & Gynecology, 2012. 120(2 Part 1). doi: 10.1097/AOG.0b013e3182605bf1 22825101

[6] Kyrgiou M., et al., Adiposity and cancer at major anatomical sites: umbrella review of the literature. BMJ, 2017. 356: p. j477. doi: 10.1136/bmj.j477 28246088 PMC5421437

[7] Ryan N.A.J., et al., The proportion of endometrial cancers associated with Lynch syndrome: a systematic review of the literature and meta-analysis. Genetics in Medicine, 2019. 21(10): p. 2167–2180. doi: 10.1038/s41436-019-0536-8 31086306 PMC8076013

[8] Mahdy H., Casey M., and Crotzer D., Endometrial Cancer, in StatPearls [Internet]. 2022, StatPearls Publishing: Treasure Island (FL). 30252237

[9] Sun D., et al., Why 90% of clinical drug development fails and how to improve it? Acta Pharm Sin B, 2022. 12(7): p. 3049–3062. doi: 10.1016/j.apsb.2022.02.002 35865092 PMC9293739

[10] Seruga B., et al., Failures in Phase III: Causes and Consequences. Clin Cancer Res, 2015. 21(20): p. 4552–60. doi: 10.1158/1078-0432.CCR-15-0124 26473191

[11] Zitter R., Chugh R.M., and Saha S., Patient Derived Ex-Vivo Cancer Models in Drug Development, Personalized Medicine, and Radiotherapy. Cancers (Basel), 2022. 14(12). doi: 10.3390/cancers14123006 35740672 PMC9220792

[12] Templeton A.R., et al., Patient-Derived Explants as a Precision Medicine Patient-Proximal Testing Platform Informing Cancer Management. Front Oncol, 2021. 11: p. 767697. doi: 10.3389/fonc.2021.767697 34988013 PMC8721047

[13] Powley I.R., et al., Patient-derived explants (PDEs) as a powerful preclinical platform for anti-cancer drug and biomarker discovery. Br J Cancer, 2020. 122(6): p. 735–744. doi: 10.1038/s41416-019-0672-6 31894140 PMC7078311

[14] Kokkinos J., et al., Does the Microenvironment Hold the Hidden Key for Functional Precision Medicine in Pancreatic Cancer? Cancers (Basel), 2021. 13(10). doi: 10.3390/cancers13102427 34067833 PMC8156664

[15] Janda M., et al., Complete pathological response following levonorgestrel intrauterine device in clinically stage 1 endometrial adenocarcinoma: Results of a randomized clinical trial. Gynecologic Oncology, 2021. 161(1): p. 143–151. doi: 10.1016/j.ygyno.2021.01.029 33762086

[16] Behrouzi R., et al., Baseline Serum HE4 But Not Tissue HE4 Expression Predicts Response to the Levonorgestrel-Releasing Intrauterine System in Atypical Hyperplasia and Early Stage Endometrial Cancer. Cancers, 2020. 12(2): p. 276. doi: 10.3390/cancers12020276 31979212 PMC7073190

[17] Morrison J., et al., British Gynaecological Cancer Society (BGCS) Uterine Cancer Guidelines: Recommendations for Practice. 2021. 10.1016/j.ejogrb.2021.11.423 35065448

[18] Barr C.E. and Crosbie E.J., The Mirena coil is a suitable treatment of early-stage endometrial cancer in obese women: FOR: Careful selection and monitoring is key. Bjog, 2020. 127(8): p. 1001. doi: 10.1111/1471-0528.16224 32350976

[19] Dore M., et al., Efficacy of the LNG-IUS for treatment of endometrial hyperplasia and early stage endometrial cancer: Can biomarkers predict response? Gynecol Oncol Rep, 2021. 36: p. 100732. doi: 10.1016/j.gore.2021.100732 33718563 PMC7933258

[20] Kokkinos J., et al., Ex vivo culture of intact human patient derived pancreatic tumour tissue. Scientific Reports, 2021. 11(1): p. 1944. doi: 10.1038/s41598-021-81299-0 33479301 PMC7820421

[21] Dore M., et al., Characterisation of Levonorgestrel-Resistant Endometrial Cancer Cells. Cancer Manag Res, 2021. 13: p. 7871–7884. doi: 10.2147/CMAR.S327381 34703309 PMC8523362

[22] Dore M., et al., Transcriptomic identification of differentially expressed genes in Levonorgestrel resistant endometrial cancer cell lines. Molecular Carcinogenesis, 2023. 62(7): p. 1038–1050. doi: 10.1002/mc.23544 37067396

[23] Centenera M.M., et al., Evidence for efficacy of new Hsp90 inhibitors revealed by ex vivo culture of human prostate tumors. Clin Cancer Res, 2012. 18(13): p. 3562–70. doi: 10.1158/1078-0432.CCR-12-0782 22573351

[24] Centenera M.M., et al., A patient-derived explant (PDE) model of hormone-dependent cancer. Mol Oncol, 2018. 12(9): p. 1608–1622. doi: 10.1002/1878-0261.12354 30117261 PMC6120230

[25] Dean J.L., et al., Therapeutic response to CDK4/6 inhibition in breast cancer defined by ex vivo analyses of human tumors. Cell cycle (Georgetown, Tex.), 2012. 11(14): p. 2756–2761. doi: 10.4161/cc.21195 22767154 PMC3409015

[26] Abreu S., et al., Patient-derived ovarian cancer explants: preserved viability and histopathological features in long-term agitation-based cultures. Scientific Reports, 2020. 10(1): p. 19462. doi: 10.1038/s41598-020-76291-z 33173111 PMC7655823

[27] Collins A., et al., Development of a patient-derived explant model for prediction of drug responses in endometrial cancer. Gynecologic Oncology, 2021. 160(2): p. 557–567. doi: 10.1016/j.ygyno.2020.11.033 33309417

[28] Karekla E., et al., Ex Vivo Explant Cultures of Non-Small Cell Lung Carcinoma Enable Evaluation of Primary Tumor Responses to Anticancer Therapy. Cancer Res, 2017. 77(8): p. 2029–2039. doi: 10.1158/0008-5472.CAN-16-1121 28202521

[29] Jamaluddin M.F.B., et al., Proteomic and functional characterization of intra-tumor heterogeneity in human endometrial cancer. Cell Reports Medicine, 2022. doi: 10.1016/j.xcrm.2022.100738 36103879 PMC9512672

[30] Xu M., et al., Targeting the tumor stroma for cancer therapy. Molecular Cancer, 2022. 21(1): p. 208. doi: 10.1186/s12943-022-01670-1 36324128 PMC9628074

[31] Ciucci A., et al., Preclinical models of epithelial ovarian cancer: practical considerations and challenges for a meaningful application. Cellular and Molecular Life Sciences, 2022. 79(7): p. 364. doi: 10.1007/s00018-022-04395-y 35705879 PMC9200670

[32] Kitson S.J. and Crosbie E.J., Endometrial cancer and obesity. The Obstetrician & Gynaecologist, 2019. 21(4): p. 237–245.

[33] Parkash V., et al., Committee Opinion No. 631: Endometrial Intraepithelial Neoplasia. Obstet Gynecol, 2015. 126(4): p. 897. 10.1097/AOG.0000000000001071 26393443

[34] Whitaker K., Earlier diagnosis: the importance of cancer symptoms. The Lancet Oncology, 2020. 21(1): p. 6–8. doi: 10.1016/S1470-2045(19)30658-8 31704136

[35] Makena M.R., et al., Cancer stem cells: Road to therapeutic resistance and strategies to overcome resistance. Biochimica et Biophysica Acta (BBA)—Molecular Basis of Disease, 2020. 1866(4): p. 165339. doi: 10.1016/j.bbadis.2018.11.015 30481586

[36] Tan B.T., et al., The cancer stem cell hypothesis: a work in progress. Lab Invest, 2006. 86(12): p. 1203–7. doi: 10.1038/labinvest.3700488 17075578

[37] Cojoc M., et al., A role for cancer stem cells in therapy resistance: cellular and molecular mechanisms. Semin Cancer Biol, 2015. 31: p. 16–27. doi: 10.1016/j.semcancer.2014.06.004 24956577

[38] Li Y., et al., Drug resistance and Cancer stem cells. Cell Communication and Signaling, 2021. 19(1): p. 19. doi: 10.1186/s12964-020-00627-5 33588867 PMC7885480

[39] Morrison H., Chapter 5—Aldehyde dehydrogenase, in Enzyme Active Sites and their Reaction Mechanisms, Morrison H., Editor. 2021, Academic Press. p. 21–26.

[40] Yue H., et al., ALDH1A1 in Cancers: Bidirectional Function, Drug Resistance, and Regulatory Mechanism. Frontiers in Oncology, 2022. 12. doi: 10.3389/fonc.2022.918778 35814382 PMC9256994

[41] Prah A., et al., How Monoamine Oxidase A Decomposes Serotonin: An Empirical Valence Bond Simulation of the Reactive Step. The Journal of Physical Chemistry B, 2020. 124(38): p. 8259–8265. doi: 10.1021/acs.jpcb.0c06502 32845149 PMC7520887

[42] Buckholtz J.W. and Meyer-Lindenberg A., MAOA and the neurogenetic architecture of human aggression. Trends in neurosciences, 2008. 31(3): p. 120–129. doi: 10.1016/j.tins.2007.12.006 18258310

[43] Sun X., et al., The MAOA Gene Influences the Neural Response to Psychosocial Stress in the Human Brain. Frontiers in Behavioral Neuroscience, 2020. 14. doi: 10.3389/fnbeh.2020.00065 32499684 PMC7243356

[44] Uzbekov M.G., Monoamine Oxidase as a Potential Biomarker of the Efficacy of Treatment of Mental Disorders. Biochemistry (Moscow), 2021. 86(6): p. 773–783. doi: 10.1134/S0006297921060146 34225599

[45] Liao C.P., et al., Loss of MAOA in epithelia inhibits adenocarcinoma development, cell proliferation and cancer stem cells in prostate. Oncogene, 2018. 37(38): p. 5175–5190. doi: 10.1038/s41388-018-0325-x 29844571 PMC7500062

[46] He Z., He J., and Xie K., KLF4 transcription factor in tumorigenesis. Cell Death Discovery, 2023. 9(1): p. 118. doi: 10.1038/s41420-023-01416-y 37031197 PMC10082813

[47] Penke L.R., et al., KLF4 is a therapeutically tractable brake on fibroblast activation that promotes resolution of pulmonary fibrosis. JCI Insight, 2022. 7(16). doi: 10.1172/jci.insight.160688 35852857 PMC9462506

[48] Di Giammartino D.C., et al., KLF4 is involved in the organization and regulation of pluripotency-associated three-dimensional enhancer networks. Nature Cell Biology, 2019. 21(10): p. 1179–1190. doi: 10.1038/s41556-019-0390-6 31548608 PMC7339746

[49] Danková Z., et al., Methylation status of KLF4 and HS3ST2 genes as predictors of endometrial cancer and hyperplastic endometrial lesions. Int J Mol Med, 2018. 42(6): p. 3318–3328. doi: 10.3892/ijmm.2018.3872 30221668 PMC6202087

