# Supplementary material for: Development of a long term, ex vivo, patient-derived explant model of endometrial cancer
Source: PLoS One. 2024 Apr 18;19(4):e0301413. doi: 10.1371/journal.pone.0301413 (PMC11025966; doi:10.1371/journal.pone.0301413)
Supplement: S1 Table — (PDF) [file pone.0301413.s001.pdf]

S1 Table. Materials Information.

Item Name	Distributor	Catalogue Number
Bond Primary Antibody Diluent	Leica Biosystems	AR9352
Bovine Serum Albumin	pH Scientific	PH100-100g
BrdU Substrate	Thermo Fisher Scientific	B23151
DAB substrate	Abcam	ab64238
DAPI mounting medium	Thermo Fisher Scientific	P36935
DNase I	Sigma-Aldrich	DN25-100mg
Gelatin Sponges	Johnson & Johnson	JJ-12505
Gibco™ DMEM/F12 plus glutamax	Thermo Fisher Scientific	10-565-018
Gibco™ Fetal Bovine Serum	Fisher Scientific	10-091-148
Gibco™ Penicillin/Streptomycin	Fisher Scientific	15-140-122
Haematoxylin Gills III	Sigma-Aldrich	1051740500
Human insulin	Sigma-Aldrich	I9278
Hydrocortisone	Sigma-Aldrich	H0888
H_2O_2	Thermo Fisher Scientific	BSPA5.500
Levonorgestrel	Sapphire Bioscience	S1727
$MgCl_2$	Sigma-Aldrich	M1028-100ML
Microscope slide	Trajan	472042491
Normal Goat Serum	Ngaio Diagnostics	X090710-8
NuPAGE™ LDS Sample Buffer	Thermo Fisher Scientific	NP0007
Paraformaldehyde	Alfa Aesar	J19943.K2
Pierce™ Dithiothreitol	Thermo Fisher Scientific	20290
Pierce™ Protease Inhibitor	Thermo Fisher Scientific	A32953
Pierce™ Chemiluminescent Substrate	Thermo Fisher Scientific	34577
RIPA Lysis and Extraction Buffer	Thermo Fisher Scientific	89901
Tris-Buffered Saline	Thermo Fisher Scientific	28358
Triton X-100	Sigma-Aldrich	X100-100ML
4-20% Tris-Glycine Gel	Thermo Fisher Scientific	XP04200BOX
