# Supplementary material for: Development of a long term, ex vivo, patient-derived explant model of endometrial cancer
Source: PLoS One. 2024 Apr 18;19(4):e0301413. doi: 10.1371/journal.pone.0301413 (PMC11025966; doi:10.1371/journal.pone.0301413)
Supplement: S2 Table — (PDF) [file pone.0301413.s002.pdf]

S2 Table. Antibody and Immunohistochemistry Information.

Western Blot Antibodies

**Antibody Name**	**Distributor**	**Catalogue number**	**Clone**	**Dilution**
γ-H2A.X	Thermo Fisher Scientific	MA1-2022	3F2	1:1000
α-tubulin	Abcam	ab7291	DM1A	1:5000

Immunohistochemistry Antibodies

**Antibody Name**	**Distributor**	**Catalogue number**	**Clone**	**Dilution**	**Detection Distributor (Cat#)**	**HIER pH / temperature (°C) / time(min)**	**Antibody time (min)**	**Control Tissue**
Proliferation/Apoptosis								
Ki67	Ventana Medical Systems	790-4286	30-9	RTU	Ventana (760-500)	CC1 / 100 / 32	16	Human tonsil
PHH3	Cell Marque	369A-15	polyclonal	1:50	Ventana (760-500)	CC1 / 95 / 36	16	Human tonsil
CC3	Cell Signaling Technology	9661S	Asp175	1:200	Ventana (760-500)	CC1 / 100 / 56	32	Human tonsil
PR	Roche Diagnostics	790-2223	1E2	RTU	Ventana (760-700)	CC1 / 100 / 24	8	Human tonsil
BrdU	Thermo Fisher Scientific	MA3-071	BU-1	1:1000	-	9 / 100 / 20	Overnight	EEC not treated with BrdU substrate
Goat anti-mouse secondary	Thermo Fisher Scientific	G-21040	-	1:100	-	-	-	-
TUNEL	Sigma-Aldrich	11684809910	-	1:10	-	-	60	DNAse-treated EEC
Candidate Biomarkers								
SATB2	Cell Marque	384R-15	EP281	1:100	Ventana (760-500)	CC1 / 100 / 32	32	Human tonsil
KLF4	Abcam	ab374149	EPR1959 0	1:100	Leica (DS9800)	9 / 100 / 30	15	Human tonsil
MAO-A	Santa Cruz	sc-271123	G-10	1:100	Leica (DS9800)	9 / 100 / 30	15	Human tonsil
ALDA1A1	Santa Cruz	sc-374149	B-5	1:750	Leica (DS9800)	9 / 100 / 30	30	Human tonsil
CD80	Santa Cruz	sc-376012	37711	1:100	Leica (DS9800)	9 100 / 20	45	Human tonsil

HIER: Heat induced epitope retrieval; RTU: Ready to use; EEC: Endometrioid endometrial cancer; CC1: Basic pH (exact pH is not given)
