# Supplementary material for: Development of a long term, ex vivo, patient-derived explant model of endometrial cancer
Source: PLoS One. 2024 Apr 18;19(4):e0301413. doi: 10.1371/journal.pone.0301413 (PMC11025966; doi:10.1371/journal.pone.0301413)
Supplement: S3 Table — (PDF) [file pone.0301413.s003.pdf]

S3 Table. Explant Viabilities from Quasi-resistant Tumours.

	**Treatment**	**% Viable Tumour Tissue Within Explant**
P5	D0	100
	Neg	100
	DMSO	50
	4 ng/mL LNG	20
	4 µg/mL LNG	90
P6	D0	100
	Neg	100
	DMSO	80
	4 ng/mL LNG	100
	4 µg/mL LNG	90
P7	D0	100
	Neg	80
	DMSO	100
	4 ng/mL LNG	100
	4 µg/mL LNG	90
