# Supplementary figures and images for: Development of a long term, ex vivo, patient-derived explant model of endometrial cancer
Source: PLoS One. 2024 Apr 18;19(4):e0301413. doi: 10.1371/journal.pone.0301413 (PMC11025966; doi:10.1371/journal.pone.0301413)

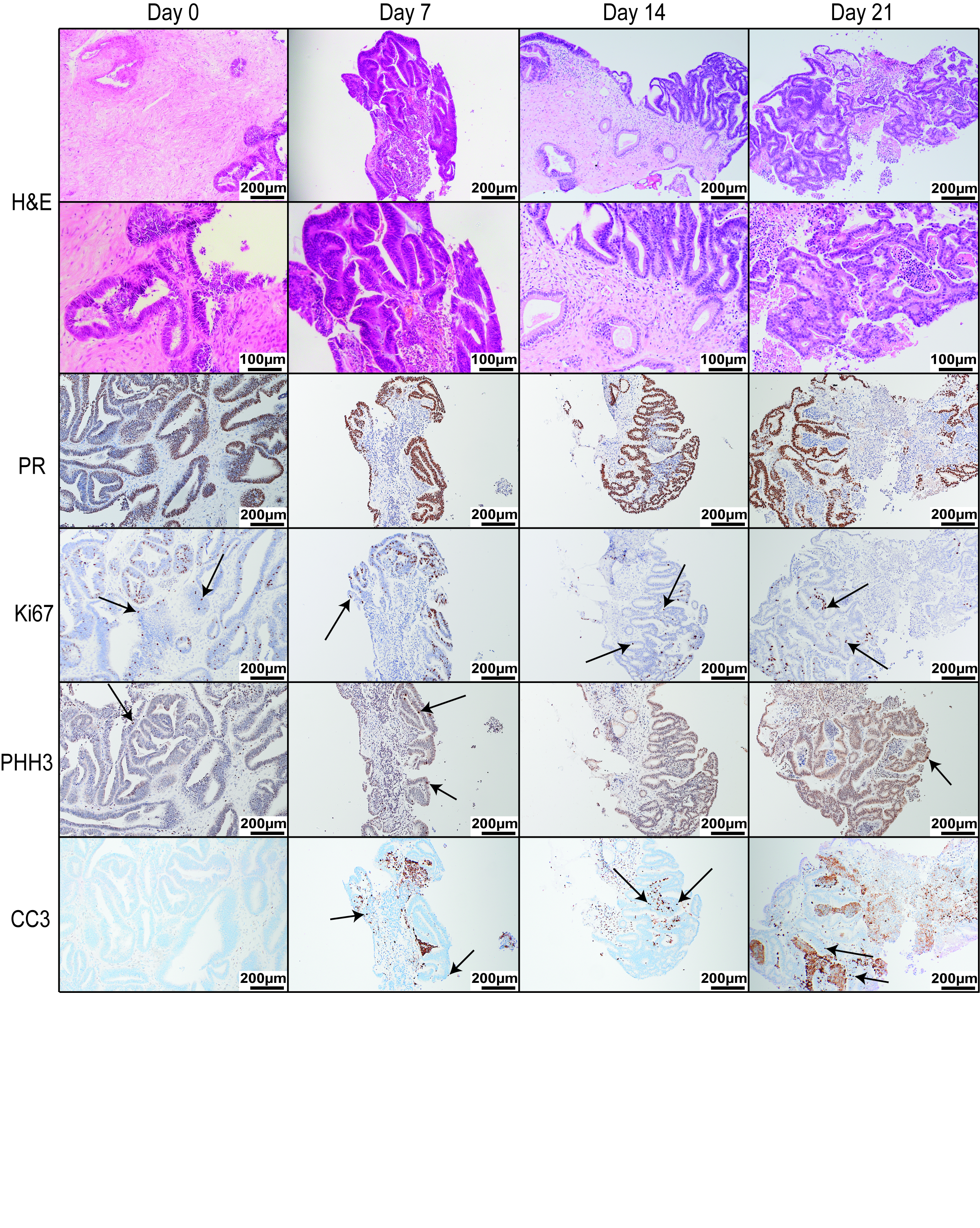

Supplement: S1 Fig — H&E and IHC for PR expression, proliferation (Ki67, PHH3) and apoptosis (CC3). H&E, haematoxylin and eosin; IHC, immunohistochemistry; PR, Progesterone Receptor; PHH3, Phosphohistone H3; CC3, Cleaved Caspase 3. (TIF) [file pone.0301413.s004.tif]

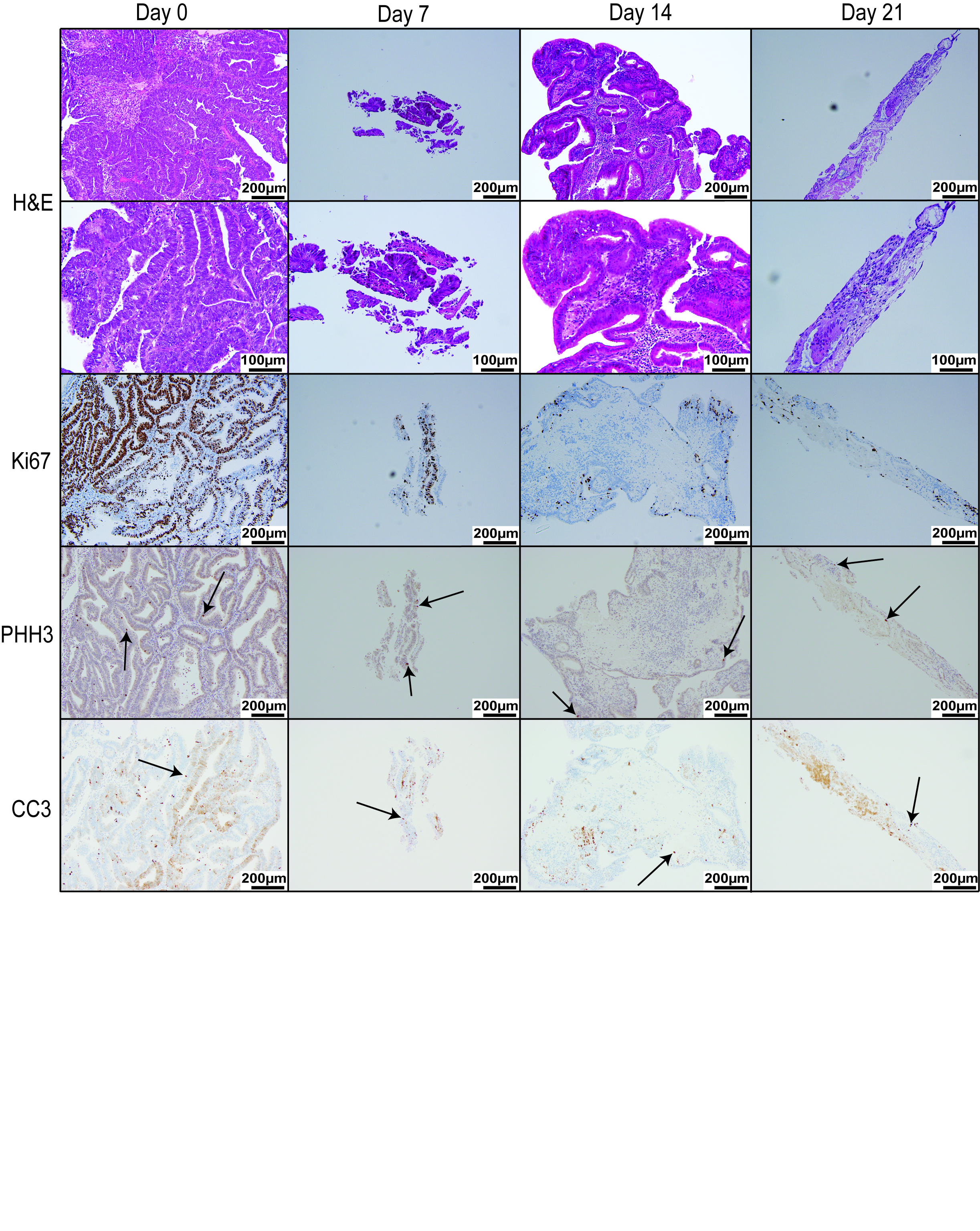

Supplement: S2 Fig — H&E and IHC for proliferation (Ki67, PHH3) and apoptosis (CC3). H&E, haematoxylin and eosin; IHC, immunohistochemistry; PR, Progesterone Receptor; PHH3, Phosphohistone H3; CC3, Cleaved Caspase 3. (TIF) [file pone.0301413.s005.tif]

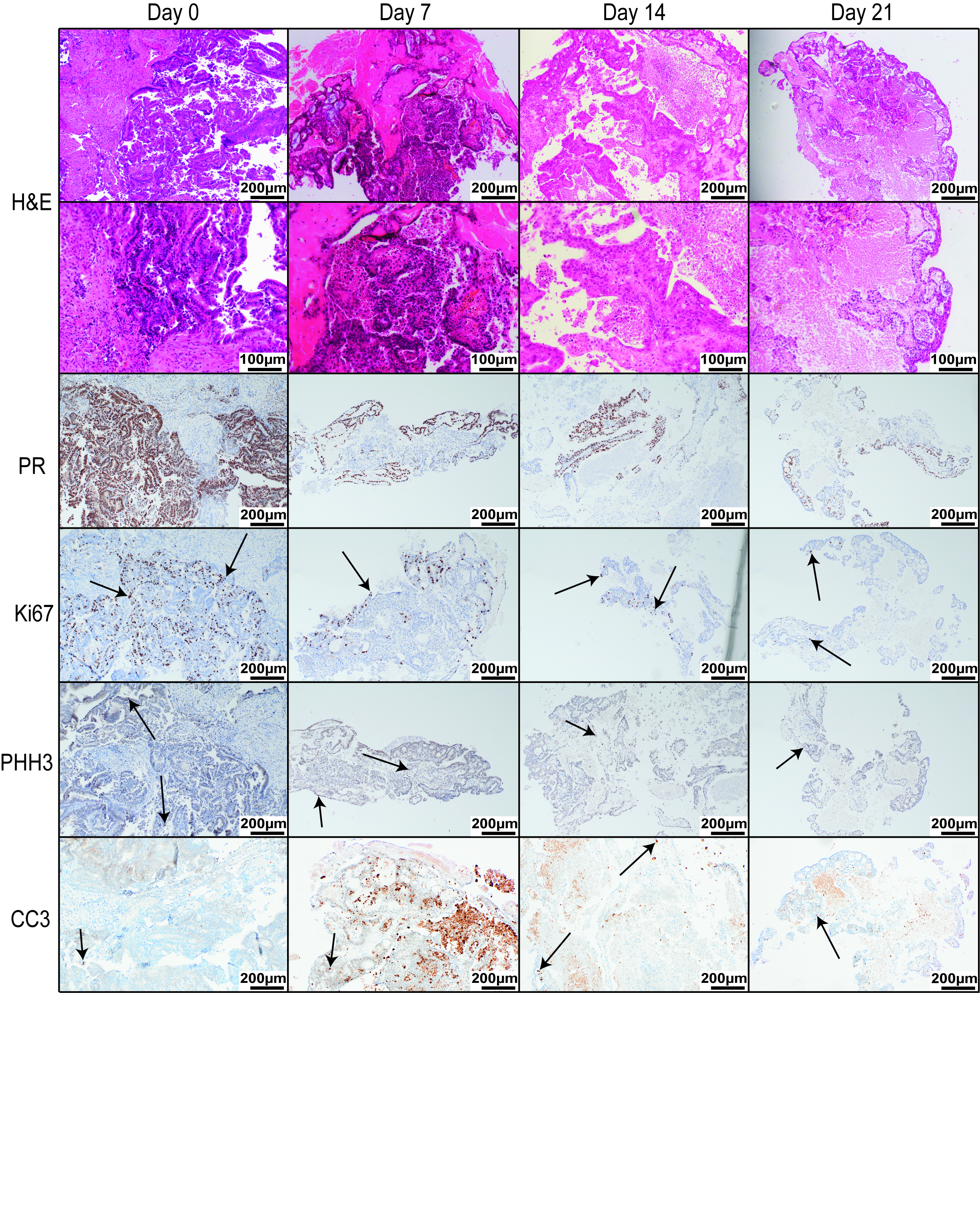

Supplement: S3 Fig — H&E and IHC for PR expression, proliferation (Ki67, PHH3) and apoptosis (CC3). H&E, haematoxylin and eosin; IHC, immunohistochemistry; PR, Progesterone Receptor; PHH3, Phosphohistone H3; CC3, Cleaved Caspase 3. (TIF) [file pone.0301413.s006.tif]

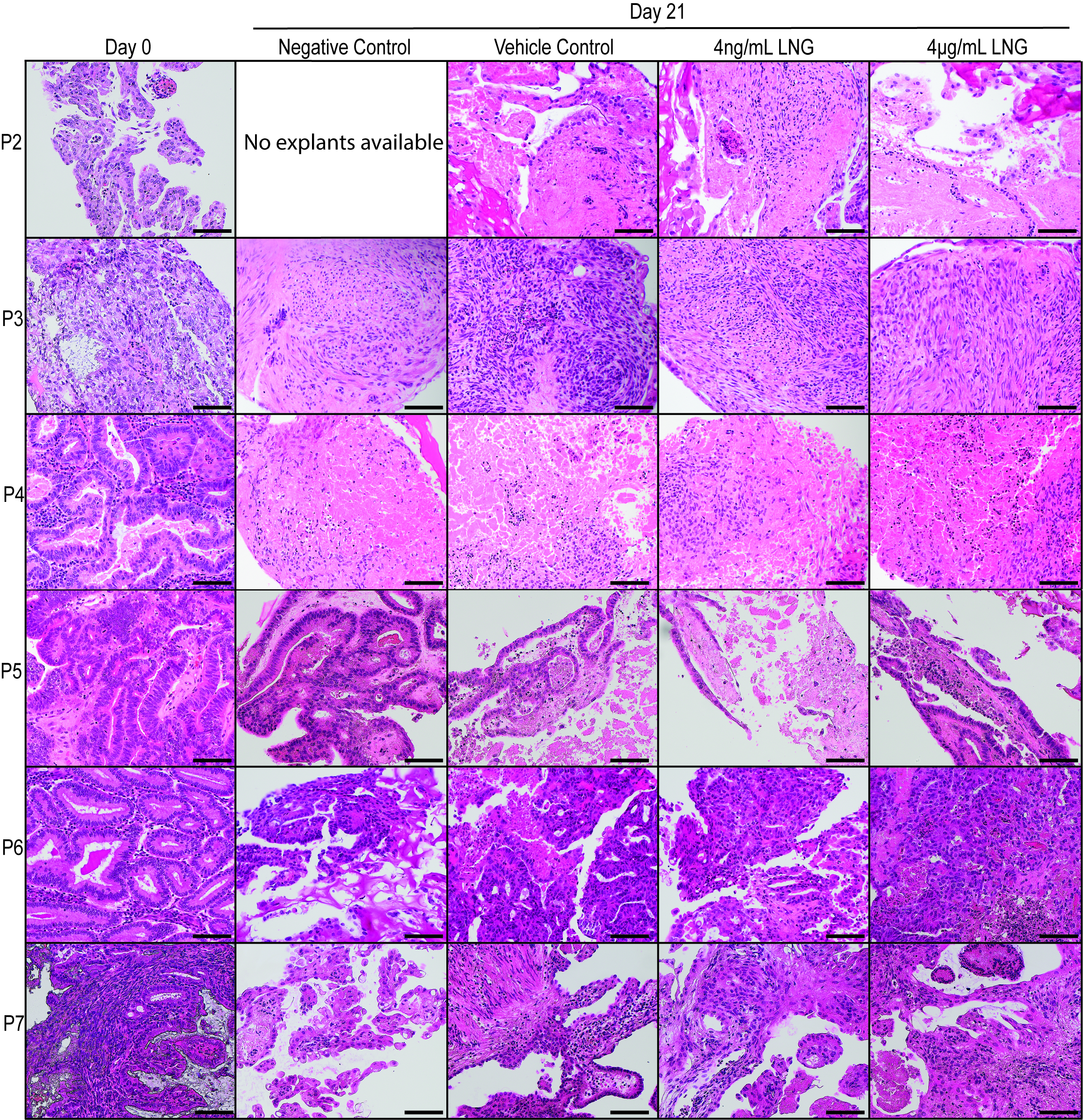

Supplement: S4 Fig — H&E was performed on tissue from three patients (P5, P6, P7) at Day 0 and Day 21 after culture with either vehicle control (culture media plus 0.08% DMSO) or LNG treatment. Scale bars represent 200 μm. H&E, Haematoxylin and Eosin; LNG, Levonorgestrel. (TIF) [file pone.0301413.s007.tif]

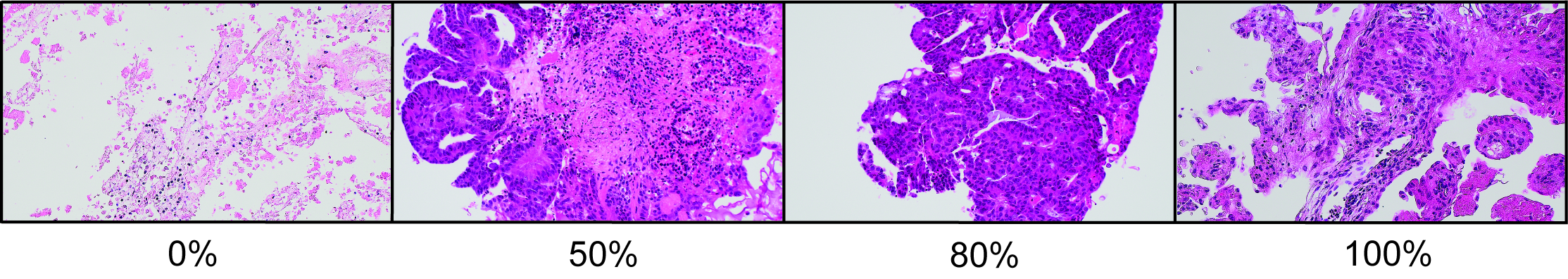

Supplement: S5 Fig — Representative images depicting explants with tumour tissue that is 0%, 50%, 80% and 100% viable as identified by a trained gynaecological histopathologist. (TIF) [file pone.0301413.s008.tif]

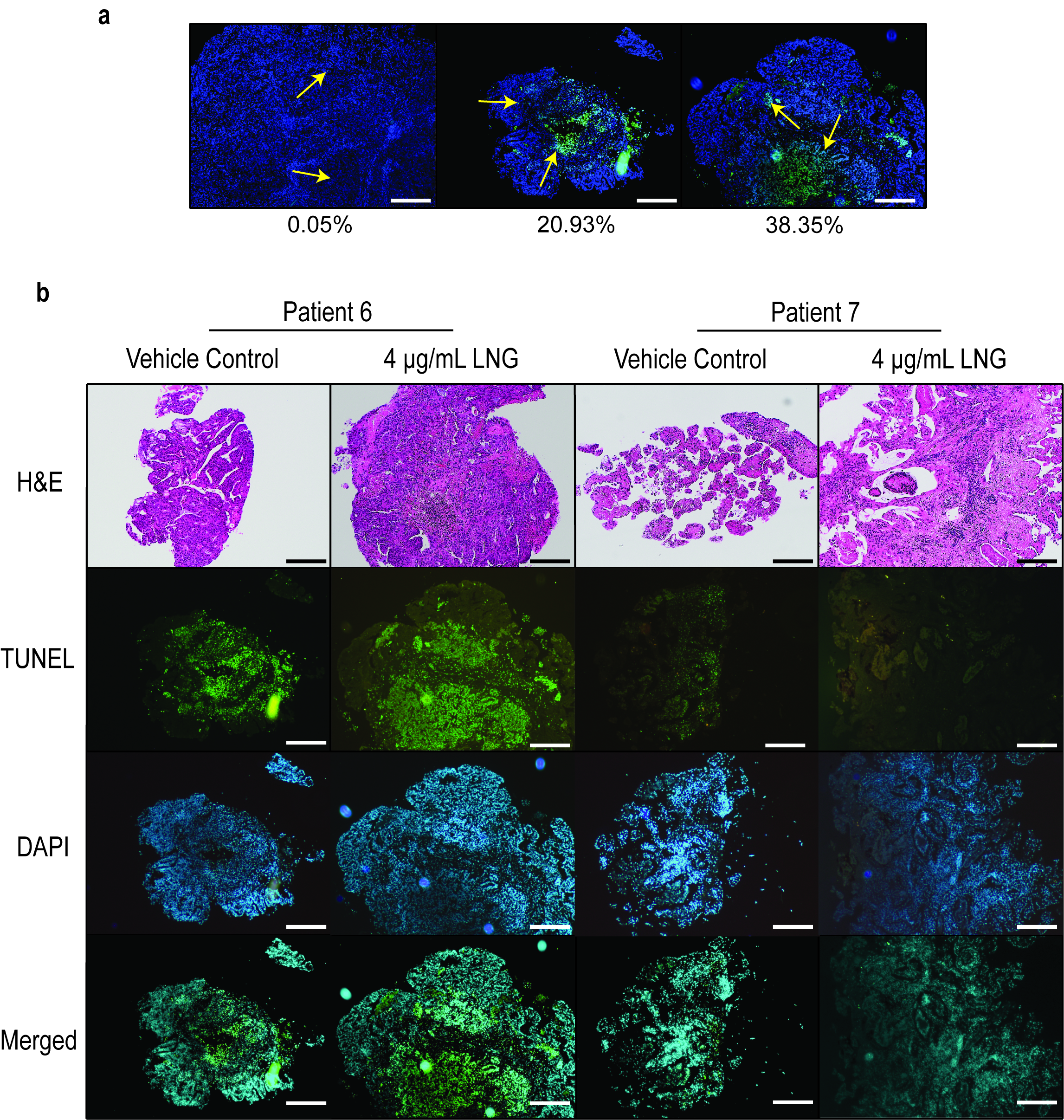

Supplement: S6 Fig — (A) Representative images for TUNEL quantification using QuPath software version 0.3.2. (B) Explants from Patients 6 and 7 which were treated with either vehicle control (culture media plus 0.08% DMSO) or 4 ug/mL levonorgestrel (LNG) for 21 days and stained with TUNEL (green) and DAPI (blue). Scale bars represent 200 μm. (TIF) [file pone.0301413.s009.tif]
