# Supplementary material for: Development of a long term, ex vivo, patient-derived explant model of endometrial cancer
Source: PLoS One. 2024 Apr 18;19(4):e0301413. doi: 10.1371/journal.pone.0301413 (PMC11025966; doi:10.1371/journal.pone.0301413)
Supplement: S1 Methods — (PDF) [file pone.0301413.s010.pdf]

S1 Methods. Levonorgestrel Calculation.

The LNG concentration used for *ex vivo* culture was calculated based on approximations gleaned from previous publications. Firstly, the uterine cavity is prolate ellipsoid in shape (1). Secondly, the average values required to determine the surface area of the endometrium are length (a), width (b) and anteroposterior depth (c) (40 mm, 38 mm and 12.61 mm, respectively) (2). These values were inputted into the Knud Thomsen's Formula for ellipsoid shapes as follows:

$$Surface\ Area = 4 \cdot \pi \left(\frac{a^p b^p + a^p c^p + b^p c^p}{3} \right)$$

Where ab, ac and bc are the distances from the shape's origin (centre) to its surface and p = 1.6075. This gave an average endometrial surface area of 2873.7 mm^2. The third approximation, the average thickness of the endometrium for stage 1A patients, is 20 mm, in line with the findings of Bahamondes *et al* (3). The capacity of endometrium receiving LNG from an IUS was therefore calculated as 57,474 mm^3. The LNG-IUS releases 20 µg of LNG per day. To calculate LNG exposure per 1 mm^3 tissue, 20 µg was divided by the endometrial capacity of 57,474 mm^3, which gave 0.000348 µg of LNG per mm^3. For standardization, this number was multiplied by three to account for the explants being up to 3 mm^3 in size, to derive a final concentration of 0.001 µg (1 ng) of LNG per explant. The final concentration of 4 ng/mL LNG accounted for each well containing up to two explants, and the volume of media in each well (500 µL). To ensure efficacy of treatment, 4 µg/mL LNG (1000x) was also tested.

References

1. Preoperative Sonographic Estimation of Uterine Volume: An Aid to Determine the Route of Hysterectomy. Journal of Gynecologic Surgery. 2002;18(1):13-22.
2. Goldstuck ND. Dimensional analysis of the endometrial cavity: how many dimensions should the ideal intrauterine device or system have? Int J Womens Health. 2018;10:165-8.
3. Bahamondes L, Ribeiro-Huguet P, Cursino de Andrade K, Leon-Martins O, Petta CA. Levonorgestrel-releasing intrauterine system (Mirena®) as a therapy for endometrial hyperplasia and carcinoma. Acta Obstetricia et Gynecologica Scandinavica. 2003;82(6):580-2.
