# Supplementary material for: Development of a long term, ex vivo, patient-derived explant model of endometrial cancer
Source: PLoS One. 2024 Apr 18;19(4):e0301413. doi: 10.1371/journal.pone.0301413 (PMC11025966; doi:10.1371/journal.pone.0301413)
Supplement: S2 Methods — (PDF) [file pone.0301413.s011.pdf]

S2 Methods. BrdU Immunohistochemistry.

10 µM Bromodeoxyuridine BrdU substrate (Thermo Fisher Scientific; MA, USA) was added to explant cultures 24 h prior to fixation at Day 21 and BrdU staining on explant sections was performed manually. Wash steps were 3 min in PBS unless otherwise stated. Slides were baked at 65°C for 45 min and rehydrated by washing twice in xylene (5 min) and twice in 100% ethanol (5 min). Heat induced epitope retrieval (HIER) was performed on a Leica Bond RX (Leica Biosystems; Germany) in 10 mM Tris-EDTA with 0.05% Tween-20. Endogenous peroxidase activity was blocked for 10 min at RT in peroxidase blocking solution containing 1% H_2O_2 (Thermo Fisher Scientific; MA, USA) and 1% methanol, and washed twice. Tissue sections were permeabilised for 15 min at RT using 0.5% triton X-100 (Sigma-Aldrich; MO, USA) dissolved in PBS and washed twice. Sections were treated with 3 U/mL DNase I (Sigma-Aldrich; MO, USA) for 1 h at 37°C and washed three times. Blocking was accomplished by addition of 10% normal goat serum (Ngaio Diagnostics) diluted in PBS for 1 h at room temp. Slides were incubated with anti-BrdU antibody (Thermo Fisher Scientific; MA, USA) overnight at 4°C, and for 30 min at RT the following day. Slides were washed three times for 5 min each (PBS and 0.05% Tween-20) and incubated with 1:100 dilution of horseradish peroxidase (HRP)-conjugated goat anti-mouse secondary antibody (Thermo Fisher Scientific; MA, USA) for 1 h at RT, and washed three times for 5 min with wash buffer. Visualisation was performed using 3,3′ diaminobenxidine (DAB) substrate (Abcam; UK) for up to 10 min. The DAB reaction was stopped by washing once for 5 min in wash buffer. Slides were treated with 1% copper sulphate for 10 min and counterstained using haematoxylin Gills III (Sigma-Aldrich; MO, USA) for 30 seconds and blued in running tap water for 10 min. Dehydration for mounting occurred in the following solutions for 2 min each: 100% ethanol twice and xylene twice. Coverslips were mounted using DPX mounting medium and tissues were analysed using a light microscope.
