# Supplementary material for: Development of a long term, ex vivo, patient-derived explant model of endometrial cancer
Source: PLoS One. 2024 Apr 18;19(4):e0301413. doi: 10.1371/journal.pone.0301413 (PMC11025966; doi:10.1371/journal.pone.0301413)
Supplement: S3 Methods — (PDF) [file pone.0301413.s012.pdf]

S3 Methods. TUNEL Immunohistochemistry.

Terminal deoxynucleotidyl transferase dUTP nick end labelling (TUNEL) was performed according to the manufacturer's instructions (Sigma-Aldrich; MO, USA). Briefly, TUNEL staining was performed after first baking, deparaffinising and rehydrating sections as described above. Slides were permeabilized by incubating at 37°C for 15 min in 20 µg/ml proteinase K dissolved in 10 mM Tris-HCl pH 7.4. Slides were washed twice with PBS. The positive control was treated with a DNAse solution (1000 U/mL DNase I, 50 mM Tris-HCl, pH 7.5, 10 mM $MgCl_2$ (Sigma-Aldrich; MO, USA)) in 1 mg/mL BSA at RT for 10 min and washed twice in PBS. TUNEL reaction mixture was prepared as outlined by the manufacturer and slides were incubated in a humidified atmosphere for 60 min in the dark at 37 °C. Labelling solution with no enzyme added was used for the negative control. Slides were rinsed twice with PBS, mounted using DAPI mounting medium (Thermo Scientific; MA, USA) and analysed using a fluorescent microscope.
